# CoCoChain: A Concept-Aware Consensus Protocol for Secure Sensor Data Exchange in Vehicular Ad Hoc Networks

**DOI:** 10.3390/s25196226

**Published:** 2025-10-08

**Authors:** Rubén Juárez, Ruben Nicolas-Sans, José Fernández Tamames

**Affiliations:** School of Engineering, Science, and Technology, UNIE Universidad, Calle Arapiles, 14, 28015 Madrid, Spain; ruben.nicolas@universidadunie.com (R.N.-S.); jose.fernandezt@universidadunie.com (J.F.T.)

**Keywords:** VANET, blockchain consensus, concept-aware protocols, sparse autoencoders, continuous concept mixing, internet of vehicles

## Abstract

Vehicular Ad Hoc Networks (VANETs) support safety-critical and traffic-optimization applications through low-latency, reliable V2X communication. However, securing integrity and auditability with blockchain is challenging because conventional BFT-style consensus incurs high message overhead and latency. We introduce CoCoChain, a concept-aware consensus mechanism tailored to VANETs. Instead of exchanging full payloads, CoCoChain trains a sparse autoencoder (SAE) offline on raw message *payloads* and encodes each message into a low-dimensional concept vector; only the top-*k* activations are broadcast during consensus. These compact semantic digests are integrated into a practical BFT workflow with per-phase semantic checks using a cosine-similarity threshold θ=0.85 (calibrated on validation data to balance detection and false positives). We evaluate CoCoChain in OMNeT++/SUMO across urban, highway, and *multi-hop broadcast under congestion* scenarios, measuring latency, throughput, packet delivery ratio, and *Age of Information* (*AoI*), and including adversaries that inject *semantically corrupted concepts* as well as cross-layer stress (RF jamming and timing jitter). Results show CoCoChain reduces consensus message overhead by up to 25% and confirmation latency by 20% while maintaining integrity with up to 20% Byzantine participants and improving information freshness (AoI) under high channel load. This work focuses on OBU/RSU semantic-aware consensus (not 6G joint sensing or multi-base-station fusion). The code, configs, and an anonymized synthetic replica of the dataset will be released upon acceptance.

## 1. Introduction

Vehicular Ad Hoc Networks (VANETs) have become a foundational component of intelligent transportation systems (ITSs), enabling direct vehicle-to-vehicle (V2V) and vehicle-to-infrastructure (V2I) communication to support both safety-critical applications—such as collision avoidance, cooperative adaptive cruise control, and real-time hazard notifications—and traffic efficiency services, including traffic signal coordination and infotainment delivery [1,2,3]. In these networks, each vehicle is typically equipped with an On-Board Unit (OBU) that periodically broadcasts status beacons (e.g., position, speed, and heading), while Roadside Units (RSUs) aggregate events and disseminate alerts or policy updates [4].

Such applications impose stringent performance constraints: end-to-end latency below 100 ms, packet delivery ratios exceeding 99%, and cryptographic assurances of authenticity and integrity—despite high mobility, frequent topology changes, and intermittent connectivity [5,6]. Furthermore, VANETs are exposed to diverse adversarial threats, including Sybil attacks, replay injection, and semantic tampering, which exacerbate reliability and trust concerns [7]. While PKI-based approaches offer cryptographic guarantees, the overhead of repeated signature verifications and certificate exchanges often overwhelms resource-constrained OBUs and RSUs [8].

To address integrity and auditability requirements, blockchain-based consensus protocols have been proposed to provide tamper-evident logging of safety-critical messages. However, conventional schemes—such as Proof-of-Work (PoW), Practical Byzantine Fault Tolerance (PBFT), or Proof-of-Stake (PoS)—incur high communication overhead and multi-phase delays, rendering them unsuitable for sub-100 ms latency demands in dense, fast-changing VANET topologies [9,10]. Even domain-specific adaptations—e.g., geographically partitioned ledgers or hierarchical overlays—struggle under congestion due to inter-domain synchronization complexity [11].

Recent advances in representation learning suggest a complementary path: replacing verbose transaction payloads with low-dimensional semantic abstractions. In particular, the Continuous Concept Mixing (CoCoMix) paradigm shows that sparse autoencoders (SAEs)—trained offline on raw message payloads—can distill sequences into interpretable concept vectors, reducing token/bandwidth load while preserving semantic fidelity [12,13]. Although conceived for language models, semantic-aware compression aligns well with time-critical, bandwidth-constrained consensus in VANETs.

Motivated by these insights, we propose CoCoChain, a concept-aware consensus protocol tailored for vehicular environments. CoCoChain leverages a sparse autoencoder to map each transaction payload to a *k*-sparse concept vector; rather than exchanging full payloads, nodes broadcast only the top-*k* activated components—the most salient semantic features—during consensus. These compact digests are embedded into a PBFT-style workflow with per-phase semantic checks, using a cosine similarity threshold θ=0.85 calibrated on a validation set to balance detection sensitivity and false positives. This design yields up to a 25% reduction in consensus communication overhead and a 20% decrease in confirmation latency, without compromising data integrity or auditability.

Beyond standard urban/highway settings, we explicitly evaluate multi-hop broadcast under congestion—where contention and collisions dominate performance—and incorporate *Age of Information introduccion-IA* (*AoI*) to capture information freshness at commit time. We discuss implications for distributed scheduling in NR-V2X Mode 2 and stress the stack with cross-layer perturbations (RF jamming and timing jitter) to probe robustness under adverse conditions [14,15].

**Contributions.** This paper makes the following contributions:

- We introduce semantic digests for VANET consensus: top-*k* concept vectors produced by an SAE and integrated into a practical PBFT workflow with per-phase semantic validation.
- We provide a latency- and bandwidth-efficient design that reduces consensus message overhead by up to 25% and confirmation latency by 20% while sustaining integrity with up to 20% Byzantine participants.
- We extend evaluation beyond benign settings to *multi-hop broadcast under congestion* and include *AoI* as a timeliness metric, alongside latency, throughput, and PDR, analyzing resilience under RF jamming and timing jitter.
- We clarify scope: this work targets OBU/RSU semantic-aware consensus and does *not* address 6G joint sensing or multi-base-station fusion.
- We commit to releasing code, configs, and an anonymized synthetic replica of the training data to support reproducibility.

## 2. Literature Review

### 2.1. Blockchain Applications in VANETs

Blockchain has been extensively investigated to strengthen integrity, auditability, and trust in Vehicular Ad Hoc Networks (VANETs), where dynamic topology and the absence of a single authority pose unique challenges. Early efforts adapted public chains directly to vehicular settings; more recent work converges toward permissioned designs and hybrid architectures tuned for stringent latency and scalability requirements [10,16,17].

Comprehensive surveys categorize vehicular blockchain solutions along three axes: (i) consensus families (e.g., PoW/PoS/BFT), (ii) permission models (public/consortium/permissioned), and (iii) target services (integrity, access control, and auditability). A consistent observation is that PoW variants are ill-suited for low-latency V2X due to energy and delay overheads, whereas BFT-style protocols can meet tighter timeliness but face scalability limits as participants increase [10,16]. These trade-offs motivate architectures that localize agreement and minimize cross-domain synchronization.

To alleviate scalability bottlenecks, several works propose *hierarchical* and *partitioned* ledgers. Representative designs include hierarchical DAG-based blockchains that maintain local chains per region plus a global chain for security, and multi-shard or graph-partitioned schemes that distribute trust storage and consensus across RSU clusters [18,19]. While such approaches improve throughput and confine failure domains, they still incur nontrivial inter-shard coordination under mobility and bursty traffic, which can stress end-to-end timeliness in dense scenarios.

A complementary line leverages *edge-assisted* deployments that offload heavy cryptographic or coordination tasks to RSUs/MEC servers while retaining decentralized guarantees. Prior work shows how combining blockchain with vehicular edge computing can reduce application-level delay when agreement is localized at the edge and global commits are amortized [20,21]. In parallel, lightweight blockchain-backed authentication schemes aim to keep join/handover costs low while preserving auditability [22].

Despite these advances, cross-domain synchronization, revocation/identity churn, and adversarial resilience in dense traffic remain open challenges highlighted repeatedly by recent surveys [10,16,17]. Our work departs from full-payload consensus by introducing *semantic digests*: top-*k* concept vectors derived offline via a sparse autoencoder and embedded into a PBFT-style workflow with per-phase semantic checks. By exchanging compact semantic evidence rather than full payloads during consensus, CoCoChain targets lower message volume and faster confirmation while preserving auditability under mobility and contention.

### 2.2. Consensus Mechanisms and Security Protocols

Traditional blockchain consensus protocols face fundamental challenges in latency-sensitive VANETs. Proof-of-Work (PoW), introduced by Nakamoto, provides probabilistic finality tied to block discovery and fork resolution; confirmation delays are inherently variable and hinge on difficulty and network propagation, making PoW mismatched with sub-100 ms vehicular safety requirements [23].

Proof-of-Stake (PoS) variants improve energy efficiency, but committee sampling, leader election, and timeout/pacemaker logic introduce latency variability and transient reconfiguration costs that are difficult to bound under high mobility. For example, Algorand explicitly targets confirmation latency on the order of minutes at the Internet scale, prioritizing safety over low latency; Ouroboros provides formal security but similarly relies on epoch/slot structures not tailored to sub-100 ms control loops [24,25].

Byzantine Fault Tolerant (BFT) protocols offer deterministic safety under partial synchrony with well-characterized communication patterns. PBFT achieves agreement via pre-prepare/prepare/commit and exhibits $O(n^{2})$ message complexity—reasonable for small committees but costly when validator churn is frequent [9]. Optimistic designs reduce common-case overhead: Zyzzyva employs speculation; HotStuff linearizes the commit rule into chained rounds, yielding O(n) multicast on the happy path and simplifying view changes, yet still disseminates payloads or payload digests through multiple phases [26,27].

Asynchronous BFT eliminates timing assumptions but replaces them with heavier communication substrates. HoneyBadgerBFT relies on reliable broadcast and threshold encryption; its foundations trace to Bracha’s reliable broadcast, whose echo/ready patterns amplify message fan-out—tolerable in datacenters but problematic on shared vehicular channels [28,29].

To reconcile scale with fault tolerance, sharded/committee-based designs process disjoint transaction sets in parallel. Omniledger demonstrates secure scale-out with cross-shard atomic commits; RapidChain applies full sharding (communication, computation, and storage) with inter-committee routing. These reduce per-committee load, but cross-shard coordination still introduces extra rounds and metadata exchanges that aggravate congestion under bursty vehicular broadcasts [30,31].

DAG/gossip families trade strong finality for metastable consensus. Avalanche’s repeated randomized sampling and IOTA’s Tangle decouple throughput from global total order and can achieve low latency in favorable conditions; however, both provide probabilistic (eventual) consistency, which raises safety and auditability concerns for safety-critical VANET decisions [32,33,34].

Hardware-assisted BFT (e.g., FastBFT with SGX) aggregates messages and prevents equivocation via TEEs to shrink communication, but this shifts trust to enclave security and deployment assumptions; known side channels and enclave management complexities limit suitability in adversarial roadside environments [35,36].

Cross-layer constraints in VANETs.In NR-V2X Mode 2 (out-of-coverage), vehicles perform autonomous sidelink resource selection; congested regions and groupcast/unicast patterns make channel occupancy exquisitely sensitive to message size, repetition, and multi-hop rebroadcasts [15]. At the application layer, the Age of Information (AoI) shows that timeliness deteriorates sharply when control messages inflate airtime under contention; thus, reducing bytes “on air” often improves freshness more than shaving a phase from the control plane [14]. These observations suggest that *payload volume*—not only phase count—dominates end-to-end performance in realistic VANET stacks.

Implication for our design. CoCoChain targets the communication substrate by replacing full payloads with *k*-sparse concept vectors and validating agreement with semantic digests inside a BFT-style workflow. By shrinking message size while preserving semantic fidelity, CoCoChain mitigates channel contention (NR-V2X Mode 2) and reduces AoI, without abandoning deterministic safety in the control plane.

### 2.3. Semantic Compression and Concept Modeling

Semantic compression leverages neural representation learning to transform high-dimensional data into compact, interpretable latent codes—often termed *concept vectors*. A canonical approach is the *k*-sparse autoencoder (SAE), which enforces a hard *k*-winners constraint on the hidden layer so that only the top-*k* activations remain nonzero, yielding minimal digests that retain salient features [37]. From a theoretical standpoint, identifiability results in sparse coding and overcomplete regimes (under incoherence and sparsity assumptions) explain when latent factors can be recovered by simple, neural-style iterative schemes [38].

Beyond deterministic sparsity, variational formulations promote structured and interpretable codes. β-VAE encourages factorized latent structure through a rate–distortion trade-off, while FactorVAE directly penalizes total correlation to enhance disentanglement [39,40]. These families supply practical knobs (e.g., sparsity/regularization strength) to balance reconstruction fidelity and semantic compactness.

Evidence from bandwidth- and resource-constrained settings supports the feasibility of autoencoder-based compression for time series, images, and sensor streams at the edge. In WSN/IIoT, autoencoder variants reduce transmitted data volume and energy with competitive distortion, confirming practicality on embedded platforms [41,42]. For graph-structured signals (e.g., dynamic connectivity), variational graph autoencoders extract compact node-level embeddings suitable for downstream tasks [43].

In wireless systems, *semantic communication* goes further by optimizing for meaning rather than symbol accuracy. DeepSC demonstrates end-to-end semantic coding that transmits compact task-relevant representations robust to channel variability [44]. These results align with vehicular constraints: in NR-V2X Mode 2 (autonomous sidelink scheduling), airtime is tightly budgeted and highly sensitive to message size and repetitions; reducing bytes “on air” directly eases contention [15]. Likewise, *Age of Information* (*AoI*)—a freshness metric central to broadcast safety beacons—benefits when control and payload messages are shorter under congestion [14].

Implication for CoCoChain. We build on these insights by adopting top-*k* concept vectors (SAE encodings) as *semantic digests* inside a BFT-style workflow. Exchanging digests instead of full payloads decreases message size at each phase without discarding task-relevant semantics, aligning with NR-V2X Mode 2 airtime constraints and improving AoI under load, while preserving auditability through consensus.

### 2.4. Adversarial Threats to Semantic Digests

While semantic compression reduces bandwidth, it also introduces attack surfaces that must be rigorously addressed to preserve consensus integrity. We group threats into three classes and outline defenses that CoCoChain integrates across the pre-prepare, prepare, and commit phases.

- **Concept poisoning (including backdoors).**

An adversary may craft inputs so that their latent encodings yield seemingly valid concept vectors while embedding misleading semantics. Clean-label and latent-space backdoors illustrate how poisoned samples can evade standard similarity checks and remain stealthy in the encoder’s representation [45,46,47]. In wireless semantic communications, physical-layer adversarial perturbations can also distort task-level meaning without obvious symbol errors [48].

- **Concept collisions (feature interference).**

When many features share a limited latent budget, different payloads may map to overlapping top-*k* supports. Mechanistically, this relates to *superposition*, whereby sparse features are packed into fewer dimensions, trading compression for interference [49]. In practice, near-duplicate detection over binary sketches (e.g., SimHash) relies on Hamming-distance thresholds [50,51], which motivates explicit *support diversity* constraints for top-*k* concept vectors.

- **Concept drift (distribution shift).**

Non-stationary traffic, protocol updates, and sensor changes shift the data distribution over time, degrading encoder fidelity and raising both false positives/negatives. The classic streaming literature documents this phenomenon and offers detectors such as EWMA charts on error rates, adaptive windows, and two-sample tests on representation distributions [52,53,54,55]. In vehicular settings, RSUs can coordinate *federated* updates to refresh encoders without centralized data pooling [56,57].

- **Integrated defense framework.**

We incorporate four complementary layers:

- **Robust training and certification.** Denoising/contractive regularization and adversarial training harden encoders against small, structured perturbations; randomized smoothing yields instance-wise certificates on encoder stability [58,59,60,61].
- **Collision-aware screening.** Enforce a minimum Hamming distance between incoming top-*k* supports and a sliding window of recent digests; trigger *selective reveal* (payload digest check) on suspicious repeats [50,51].
- **Drift monitoring and adaptive retraining.** Continuously monitor concept distributions via EWMA, ADWIN, and kernel two-sample tests on encoder outputs; initiate RSU-led federated retraining when divergence crosses calibrated thresholds [52,53,55,56,57].
- **Multimodal authenticity.** Bind each semantic digest to a Merkle commitment of the original payload. Signatures and timestamps are verified according to C-ITS standards, ETSI TS 102 941 [62], and ETSI TS 103 759 [63]. This approach enables lightweight semantic exchange without forfeiting end-to-end auditability [64].

By combining semantic digests with certified robustness, collision-aware filters, drift-responsive retraining, and cryptographic bindings to the full payload, CoCoChain aims to preserve deterministic safety while keeping messages short enough for latency-sensitive VANET operation.

### 2.5. Positioning w.r.t. C-V2X Resource Allocation, AoI-Aware MAC, and NR-V2X Mode 2 Models

(a) Multi-agent resource allocation in C-V2X Mode 4.

Recent work frames semi-persistent scheduling in sidelink Mode 4 as a decentralized multi-agent control problem, where vehicles collaboratively select time–frequency resources to reduce collisions and improve the packet delivery ratio (PDR) [65]. These schemes optimize *who uses the channel when/where* under partial observability and interference coupling. In contrast, *CoCoChain* is orthogonal: it operates *above* the MAC as a consensus/ledger layer. Our contribution reduces per-message airtime via top-*k* semantic digests and gates malformed traffic early, thereby decreasing contention pressure that any Mode 4 allocator—learning-based or heuristic—must handle. In deployments, CoCoChain can coexist with (and benefit from) Mode 4 multi-agent schedulers: better resource allocation lowers collision/backoff bursts, while smaller CoCoChain wire images further ease the allocator’s load.

(b) AoI-aware MAC mechanisms in VANETs.

Age-of-Information (AoI)-aware congestion control explicitly targets freshness by adapting beaconing and access probability to the current channel busy ratio (CBR) and experienced delay [14]. These mechanisms act at the access layer to shrink service time and reduce queuing, thus lowering expected AoI. CoCoChain’s top-*k* concept exchange complements AoI-aware MAC in two ways: (i) by shrinking the payload from *d* floats to *k* index–value pairs (k≪d), we proportionally reduce on-air time per consensus message, improving service rate and cutting queuing delay; (ii) by filtering semantically inconsistent items before replication, we prevent wasteful retransmissions that inflate AoI during bursts. Empirically (Section 4.5, Section 4.6 and Section 4.7), this yields lower confirmation latency at a given CBR, suggesting that semantic compression can be layered with AoI-aware MAC to jointly optimize freshness and integrity.

(c) Analytical NR-V2X Mode 2 models (PDR–CBR curves).

Analytical and system-level studies of NR-V2X Mode 2 provide calibrated mappings between traffic load, CBR, and reliability (PDR) across freeway/urban channels and sensing-based resource selection [15]. We leveraged these curves to set offered-load envelopes and to validate our PHY/MAC parameterization for highway and cross-domain scenarios (e.g., path loss exponents, sensing window sizes, and target CBR ranges). Where relevant, we cross-checked our IEEE 802.11p settings against SINR-based broadcast analyses [66] and ETSI ITS-G5 guidance [67]. This grounding ensures that CoCoChain’s measured latency/throughput improvements are reported within realistic CBR and PDR regimes, not in under-loaded artifacts.

Multi-agent Mode 4 resource allocation [65] and AoI-aware MAC [14] optimize channel access and freshness at the link/MAC layer; NR-V2X Mode 2 analyses [15] and IEEE 802.11p/bd models [66] provide reliability baselines. *CoCoChain* addresses a distinct gap at the *consensus layer*: by interleaving semantic validation with PBFT and by minimizing the wire image of consensus messages, it reduces airtime and retransmissions *independently* of the specific allocator/MAC, thereby improving end-to-end finality and robustness under realistic CBR–PDR operating points. These layers are complementary and can be jointly deployed.

### 2.6. Positioning of CoCoChain

*CoCoChain* aims to integrate *semantic compression* directly within a Byzantine Fault Tolerant (BFT) consensus workflow tailored to VANETs. To the best of our knowledge, within the scope of VANET consensus, prior efforts either optimize full-payload dissemination or accelerate crypto/coordination at the edge, whereas *CoCoChain* couples *top-k concept digests* with a PBFT-style protocol to meet low-latency and auditability requirements in highly dynamic settings [9,21,27,44].

Building on *k*-sparse autoencoders [37] and recent advances in semantic communications [44], *CoCoChain* introduces the following:

- **Domain-specific concept dictionary.** A *k*-sparse SAE is trained offline on heterogeneous V2V/V2I traces (urban, highway, and cross-domain) to capture domain-relevant patterns (e.g., maneuvers, hazard types) in a *C*-dimensional latent space [37].
- **Top-*****k*** **payload encoding.** Instead of broadcasting full payloads or large digests, nodes exchange only the indices and activations of the top-*k* latent units, i.e., semantic digests ${\left\{\left(i,c_{i}\right)\right\}}_{i=1}^{k}$, thereby shrinking per-message size during each BFT phase while preserving task-relevant semantics [37,44].
- **Semantic BFT workflow.** Classical pre-prepare, prepare, and commit stages are augmented with per-phase cosine-similarity checks on semantic digests, enabling early discard/quarantine of inconsistent transactions before any payload reveal, while retaining deterministic safety guarantees [9,27].
- **Edge-assisted validation.** RSUs cache concept prototypes and perform rapid similarity lookups (e.g., GPU-enabled at the edge), offloading verification from OBUs and aligning with MEC-based V2X designs that localize agreement to reduce on-air time and contention [21].
- **Comprehensive evaluation.** We assess performance in the Veins/OMNeT++/SUMO stack across urban, highway, and multi-hop congestion scenarios, under both honest and adversarial conditions (semantic poisoning/collisions), and report latency/throughput/PDR alongside *Age of Information* (*AoI*) [4,14,15].

In contrast to hybrid designs that defer integrity/auditability to secondary ledgers or rely solely on trusted hardware to trim message complexity, *CoCoChain*’s *semantic-first* approach targets the dominant cost driver in broadcast vehicular stacks—payload volume—without abandoning BFT-style finality [35,68].

## 3. Methodology

This section describes the design of *CoCoChain*, a concept-aware consensus protocol that integrates sparse semantic encoding into a Byzantine Fault Tolerant (BFT) workflow tailored for vehicular environments. We detail the system model, the training of the sparse autoencoder (SAE), the extraction and selection of semantic concepts, and their incorporation into a PBFT-style consensus mechanism.

### 3.1. System Model and Assumptions

We consider a permissioned Vehicular Ad Hoc Network (VANET) with *n* nodes comprising On-Board Units (OBUs) on vehicles and fixed Roadside Units (RSUs). Communication uses IEEE 802.11p as the baseline PHY/MAC; sensitivity to congestion-driven broadcast and multi-hop rebroadcast is analyzed in Section 4, and we discuss implications vis-à-vis NR-V2X Mode 2 (autonomous sidelink scheduling) [15]. The network is *partially synchronous*: there exists an upper bound Δ on message delay during normal operation, while transient asynchrony periods may violate this bound [69]. Nodes keep loosely synchronized clocks (e.g., GNSS/RSU beacons) with bounded skew ε and sign application timestamps.

Each node i∈{1,…,n} maintains the following:

- An ECDSA key pair $\left({\mathrm{sk}}_{i},{\mathrm{pk}}_{i}\right)$ for signing and verification, and a cryptographic hash H(·) for payload commitments (aligned with C-ITS security services) [70].
- A locally stored sparse autoencoder $\left(f_{\theta },g_{\phi }\right)$ trained offline on heterogeneous V2V/V2I traces using reconstruction loss plus an $\ell_{1}$ sparsity term [37].
- A cosine-similarity threshold τ=0.85 to validate that received concept vectors c match locally recomputed $\hat{c}=f_{\theta }\left(x\right)$, i.e., $\cos(c,\hat{c})\geq \tau$.
- An LRU cache of size *M* with recent full payloads and their concept vectors for on-demand reveal and replay protection.

We model the time-varying communication topology as $G_{t}=\left(V,E_{t}\right)$, where *V* is the set of OBUs/RSUs and $\left(i,j\right)\in E_{t}$ if nodes *i* and *j* can exchange IEEE 802.11p beacons at time *t*. Event dissemination may traverse up to $H_{\max}$ hops (urban/highway), reflecting re-broadcast under congestion.

**Figure 1:**
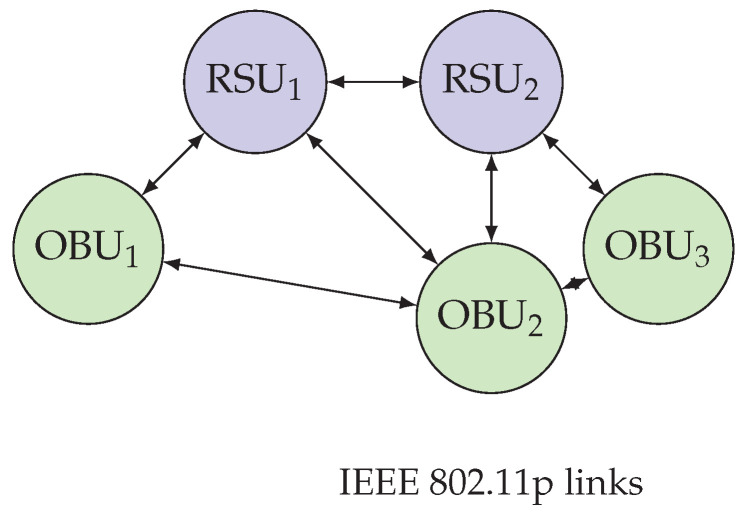
Abstract system model: OBUs and RSUs interconnected over IEEE 802.11p with partial synchrony bound Δ.

As depicted in Figure 1, we model the time-varying communication graph and multi-hop dissemination with partial synchrony.

- **Security and trust assumptions.**

Up to

$$f\;<\;\left\lfloor \frac{n}{3}\right\rfloor$$

nodes may behave in a Byzantine manner (drop/forge/replay and attempt semantic manipulation). A PKI issues and revokes certificates off-chain; misbehavior handling follows C-ITS practices (revocation lists and reporting) [63]. The adversary can increase channel contention (e.g., localized jamming) but cannot compromise more than *f* replicas.

- **Semantic digests and AoI.**

Given a payload *x* with signed timestamp ts(x), the encoder computes $z=f_{\theta }\left(x\right)$ and the top-*k* semantic digest $d=\mathrm{TopK}\left(z\right)={\left\{\left(i,c_{i}\right)\right\}}_{i=1}^{k}$. Each transaction carries a binding commitment

$$\mu \;=\;H\left(x∥ts(x)∥\mathrm{nonce}\right),$$

cryptographically tying the semantic digest to the original payload. We track *Age of Information* (AoI) as

$$\Delta_{\mathrm{AoI}}\left(t\right)\;=\;t_{\mathrm{commit}}-ts\left(x\right),$$

in addition to latency, throughput, and PDR [14].

- **Semantic-PBFT algorithm.**

We integrate semantic digests into a PBFT-style pipeline with selective reveal: the primary proposes (v,s,μ,d) and replicas verify signatures and semantic consistency, fetching the full payload only upon failure or collision suspicion [9]. Algorithm 1 outlines the local logic at node *i*.
**Algorithm 1** Semantic-PBFT at node *i*  1:**Input:** payload *x*, timestamp ts(x), threshold τ, SAE $f_{\theta }$  2:$z\leftarrow f_{\theta }\left(x\right);\;d\leftarrow \mathrm{TopK}\left(z\right);\;\mu \leftarrow H\left(x∥ts\left(x\right)∥\mathrm{nonce}\right)$  3:$\sigma \leftarrow \mathrm{Sign}({\mathrm{sk}}_{i},\langle v,s,\mu ,d\rangle )$  4:Broadcast 〈PRE_PREPARE,v,s,μ,d,σ〉  5:**for** each $\langle \mathrm{PRE\_PREPARE},v,s,\mu^{\prime },d^{\prime },\sigma^{\prime }\rangle$ from primary *j* **do**  6:      **if** $\mathrm{Verify}({\mathrm{pk}}_{j},\langle v,s,\mu^{\prime },d^{\prime }\rangle ,\sigma^{\prime })$ **then**  7:            **if** $\mathrm{cached}(\mu^{\prime })$ **then**  8:                  Retrieve $x^{\prime }$ by $\mu^{\prime }$; $\hat{z}\leftarrow f_{\theta }\left(x^{\prime }\right)$  9:                  **if** $\cos(\mathrm{vec}\left(d^{\prime }\right),\hat{z})\geq \tau$ **then**10:                      Broadcast $\langle \mathrm{PREPARE},v,s,\mu^{\prime },d^{\prime },\sigma_{i}\rangle$11:                  **else**12:                      Discard (semantic mismatch)13:                  **end if**14:            **else**15:                  **Selective reveal:** request $x^{\prime }$ by $\mu^{\prime }$; verify and proceed as above16:            **end if**17:      **else**18:            Discard (invalid signature/format)19:      **end if**20:**end for**21:**for** each 2f+1 matching PREPAREs on 〈v,s,μ,d〉 **do**22:      Broadcast $\langle \mathrm{COMMIT},v,s,\mu ,d,\sigma_{i}\rangle$23:**end for**24:**for** each 2f+1 matching COMMITS **do**25:      If *x* not cached, fetch by μ; verify and append 〈μ,d,x,ts(x)〉 to the ledger26:      Update AoI and caches27:**end for**

This design ensures that compact semantic digests traverse the network in the common case, while full payloads are revealed on demand for auditing or upon semantic mismatch. Message authentication and timestamp binding via μ align with C-ITS security services, and the partial synchrony bound Δ governs view-change timeouts.

### 3.2. Sparse Autoencoder Training

To obtain compact and semantically meaningful representations of vehicular messages, we train a *k*-sparse autoencoder (SAE) offline on a mixed dataset composed of synthetic traces generated with SUMO [71] and curated V2V/V2I logs (urban and highway). The SAE provides top-*k semantic digests* used later during consensus.

- **Data preparation.**

Each raw payload $x\in R^{d}$ concatenates position (latitude, longitude), velocity vector, heading, timestamp deltas, and one-hot event flags (e.g., braking, hazard), yielding d=64. We standardize all continuous features (zero mean, unit variance) and apply data augmentation with (i) additive Gaussian noise (σ=0.02) and (ii) random packet-drop masks (Bernoulli $p_{\mathrm{loss}}=0.1$) to mimic lossy links. Categorical flags are left as one-hot vectors. The dataset is split 80/10/10 (train/val/test) with fixed seeds to ensure reproducibility.

- **Model architecture.**

The SAE comprises an encoder $f_{\theta }:R^{d}\to R^{C}$ and a decoder $g_{\phi }:R^{C}\to R^{d}$, with C=128. The encoder uses two fully connected layers (64 → 256 → 128) with ReLU activations and batch normalization [72], followed by a hard top-*k* selection layer (with k=8) that zeroes all but the *k* largest activations [37]. The decoder mirrors the encoder (128 → 256 → 64) with ReLU and a linear output. The top-*k* operator is trained via a straight-through estimator for backpropagation through the hard selection [73].

- **Objective and regularization.**

We minimize a reconstruction-plus-sparsity objective:

$$L\left(\theta ,\phi \right)=\frac{1}{N}\sum_{i=1}^{N}{∥x_{i}-g_{\phi }\left(f_{\theta }\left(x_{i}\right)\right)∥}_{2}^{2}\;+\;\lambda \;{∥f_{\theta }\left(x_{i}\right)∥}_{1},$$

with $\lambda =10^{-3}$ encouraging sparse codes. We add an $L_{2}$ weight decay of $10^{-5}$ to all trainable parameters to mitigate overfitting.

- **Training procedure**

Parameters are initialized with Xavier uniform [74] and optimized using Adam ($\mathrm{lr}=1\times 10^{-3}$, batch size 512) [75]. We train for up to 200 epochs with early stopping (patience 10) based on validation loss and employ a cyclical learning-rate (CLR) schedule between $10^{-4}$ and $10^{-3}$ to escape shallow minima. Unless otherwise noted, results reported in Section 5 correspond to a converged model (typically ≈80 epochs) with average sparsity ≈ 8 active units per input (matching *k*).

- **Hyperparameter sensitivity.**

We grid-searched k∈{4,8,16} and $\lambda \in \{10^{-4},10^{-3},10^{-2}\}$. The setting k=8, $\lambda =1\times 10^{-3}$ offered the best compression–fidelity trade-off across urban, highway, and cross-domain splits (test MSE <0.003). Increasing the latent width to C=256 yielded only marginal reconstruction gains (<5% MSE reduction) at the cost of larger digests and higher on-air time during consensus, which contradicts our latency budget.

- **Reproducibility details.**

We will release training scripts, configuration files (including seeds, normalization statistics, and *k*, λ, and *C*), and a synthetic, anonymized replica of the training data (feature schema identical to the real logs) upon acceptance. Experiments were implemented in PyTorch 2.2.2 (Python 3.10); precise versions of all dependencies (e.g., NumPy, TorchVision, CUDA/cuDNN), RNG seeds, and hardware descriptors (GPU model and driver) will be included in a README to enable faithful reproduction.

- **Pseudo-code.**

Algorithm 2 summarizes the offline training loop.
**Algorithm 2** Offline SAE Training**Require:** Dataset ${\left\{x_{i}\right\}}_{i=1}^{N}$, sparsity *k*, sparsity weight λ
  1:Initialize (θ,ϕ) with Xavier uniform [74]  2:Optimizer ← Adam(θ,ϕ, lr = 10^−3^, weight_decay = 10^−5^) [75]  3:**for** epoch = 1 to 200 **do**  4:      **for** each minibatch $B\subset \{x_{i}\}$ **do**  5:            $z_{j}\leftarrow f_{\theta }\left(x_{j}\right)$;   ${\hat{c}}_{j}\leftarrow \mathrm{TopK}\left(z_{j},k\right)$    (STE backward) [73]  6:            ${\hat{x}}_{j}\leftarrow g_{\phi }\left({\hat{c}}_{j}\right)$  7:            $L\leftarrow \frac{1}{\left|B\right|}\sum_{j}∥{\hat{x}}_{j}-x_{j}{∥}_{2}^{2}+\lambda \;{∥z_{j}∥}_{1}$  8:            Backpropagate $\nabla_{\theta ,\phi }L$ and update  9:      **end for**10:      **if** validation loss ≮ best for 10 epochs **then**11:            **break**12:      **end if**13:**end for**


These procedures yield stable, sparse concept vectors suitable for low-latency semantic consensus while keeping digests compact for broadcast under congestion.

### 3.3. Concept Extraction and Top-k Selection

After training the sparse autoencoder (SAE), each transaction *t* with raw payload x=payload(t) undergoes a two-step process that extracts a compact, semantically salient representation used during consensus.

- **Encoding.**

The encoder $f_{\theta }:R^{d}\to R^{C}$ maps the input to a dense concept vector

$$z\;=\;f_{\theta }\left(x\right),\;z={\left[z_{1},\ldots ,z_{C}\right]}^{⊤},$$

computed with two matrix–vector multiplies and pointwise non-linearities (ReLU/BN), i.e., O(dC) FLOPs for the first affine and $O(C^{2})$ for the second in our two-layer MLP. We denote by vec(·) the canonical expansion that maps an index–value set back to $R^{C}$ (zeros elsewhere), used later in semantic checks.

- **Top-*k* selection.**

To enforce sparsity, we retain only the *k* largest-magnitude coordinates of z. Let

$$S\;=\;\left\{\;i\in \left\{1,\ldots ,C\right\}:\;|z_{i}\left|\;\mathrm{is}\;\mathrm{among}\;\mathrm{the}\;\mathrm{top}\text{-}k\;\mathrm{values}\;\mathrm{of}\;\{\right|z_{1}\left|,\ldots ,\right|z_{C}\left|\right\}\right\}.$$

We compute *S* using a fixed-size min-heap in O(Clogk) time; a partial-selection routine (e.g., Quickselect/Introselect) yields O(C) expected time if preferred.

**Determinism.** To avoid replica divergence when ties occur, we break ties by (i) larger $|z_{i}|$, then (ii) smaller index *i*. The resulting sparse digest is $d={\left\{\left(i,z_{i}\right)\right\}}_{i\in S}$.

To avoid replica divergence when ties occur, we break ties by (i) larger $|z_{i}|$, then (ii) smaller index *i*. The resulting sparse digest is the index–value set

$$d\;=\;\mathrm{TopK}\left(z,k\right)\;=\;{\left\{\left(i,\;z_{i}\right)\right\}}_{i\in S},$$

and its dense expansion is $\mathrm{vec}\left(d\right)\left[i\right]=z_{i}$ if i∈S, else 0.

- **Binary format and quantization (for broadcast).**

During consensus we broadcast only *d* (and the header/commitment), not the full payload. Indices are encoded as unsigned integers with the minimum width ($\left\lceil \log_{2}C\right\rceil$ bits); with C=128 we use one byte per index. Activations are transmitted either as 32-bit floats or as 8-bit symmetric quantized values

$${\tilde{z}}_{i}\;=\;\mathrm{round}\left(\frac{z_{i}}{s}\right),\;s=\frac{\alpha }{127},$$

with scale *s* chosen per-batch/per-message from $\alpha =\max_{i\in S}\left|z_{i}\right|$ (zero-point =0) [76]. This keeps decoding simple and preserves sign.

**Size example** If the baseline carried d=64 floats (256 B), a digest with k=8 and C=128 uses (i) 8 indices + 8 float32 values ≈8×(1+4)=40 B, i.e., ∼84% reduction; or (ii) 8 indices + 8 int8 values ≈8×(1+1)=16 B, i.e., ∼94% reduction. Smaller on-air footprints directly ease contention in autonomous sidelink scheduling (NR-V2X Mode 2) and improve freshness under load as captured by AoI [14,15].

- **Pipeline diagram.**

**Figure 2:**
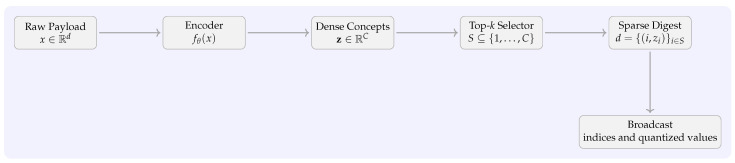
Concept extraction and top-*k* selection pipeline. The consensus path carries only indices and (optionally quantized) activations.

Figure 2 illustrates the end-to-end flow—from raw payload to the byte-packed top-*k* digest used in consensus.

Complexity and optimizations.

- **Encoding:** Two affine layers + BN/ReLU ⇒ dense linear algebra kernels; amenable to INT8 inference when using quantized weights/activations [76].
- **Selection:** O(Clogk) with a min-heap or O(C) expected with Quickselect; tie-breaking is deterministic (by $|z_{i}|$, then *i*) [77].
- **Communication:** Payload shrinks from *d* floats to *k* index–value pairs. With C≤256 we use 1 B indices; with INT8 activations, total is 2k bytes + header/commitment. Smaller digests reduce airtime and help maintain low AoI under congestion [14,15].

### 3.4. Concept-Interleaved PBFT Workflow

CoCoChain extends the classical PBFT pipeline by interleaving semantic validation at every phase while cryptographically binding each semantic digest to its original payload. Let τ denote the cosine-similarity threshold and μ=H(x∥ts(x)∥nonce) the per-transaction commitment (Section 3.1). Safety follows the standard PBFT assumptions under partial synchrony [9,69].

1. Pre-Prepare (leader).

The primary collects *m* client transactions $t_{1},\ldots ,t_{m}$ with payloads $x_{j}$ and headers $\mathrm{hdr}(t_{j})$. For each *j*, it computes:

$$z^{\left(j\right)}=f_{\theta }\left(x_{j}\right),\;d^{\left(j\right)}=\mathrm{TopK}\left(z^{\left(j\right)},k\right),\;\mu^{\left(j\right)}=H\left(x_{j}∥ts\left(x_{j}\right)∥\mathrm{nonce}\right).$$

he primary then proposes the block’s semantic summary

$$B_{\mathrm{sem}}={\left\{\;(\mathrm{hdr}\left(t_{j}\right),\;\mu^{\left(j\right)},\;d^{\left(j\right)})\;\right\}}_{j=1}^{m}$$

and broadcasts

$$\langle \mathrm{PRE\_PREPARE},\;v,\;B_{\mathrm{sem}},\;\sigma_{\ell }\rangle ,$$

where *v* is the view number and $\sigma_{\ell }$ signs $\left(v,B_{\mathrm{sem}}\right)$ per C-ITS services.

2. Prepare (replicas).

Upon a valid PRE_PREPARE, replica *i* performs per-transaction checks:

(a) **Cache path (fast):** If $x_{j}$ is locally cached by $\mu^{\left(j\right)}$, compute ${\tilde{z}}^{\left(j\right)}=f_{\theta }\left(x_{j}\right)$ and form the dense vector $\mathrm{vec}(d^{\left(j\right)})$; accept the item if
  $$s_{j}=\cos\left(\mathrm{vec}\left(d^{\left(j\right)}\right),\;{\tilde{z}}^{\left(j\right)}\right)\;\geq \;\tau .$$
(b) **No-cache path (selective reveal):** If $x_{j}$ is not cached, perform format/signature checks on $\left(\mu^{\left(j\right)},d^{\left(j\right)}\right)$, enforce $|d^{\left(j\right)}|=k$ and index ranges; if the item is flagged (e.g., repeated top-*k* support beyond expectation or inconsistent metadata), request $x_{j}$ by $\mu^{\left(j\right)}$ (*selective reveal*) and apply the cosine test above; otherwise, defer payload fetch to the commit stage.
(c) If any item fails, discard $B_{\mathrm{sem}}$ and stop processing.

If all items pass, compute $\mathrm{digest}(B_{\mathrm{sem}})$ and broadcast

$$\langle \mathrm{PREPARE},\;v,\;\mathrm{digest}\left(B_{\mathrm{sem}}\right),\;\sigma_{i}\rangle .$$

3. Commit (replicas).

After gathering 2f+1 matching PREPARE messages for $\left(v,\mathrm{digest}\left(B_{\mathrm{sem}}\right)\right)$, broadcast

$$\langle \mathrm{COMMIT},\;v,\;\mathrm{digest}\left(B_{\mathrm{sem}}\right),\;\sigma_{i}\rangle .$$

Upon receiving 2f+1 valid COMMITs, each replica performs the following:

(a) Fetches any missing payloads $x_{j}$ by $\mu^{\left(j\right)}$ (*payload reveal*); verifies signatures and recomputes $f_{\theta }\left(x_{j}\right)$ to confirm $\cos\left(\mathrm{vec}\left(d^{\left(j\right)}\right),f_{\theta }\left(x_{j}\right)\right)\geq \tau$;
(b) If all items pass, appends $\langle \mu^{\left(j\right)},d^{\left(j\right)},x_{j},ts\left(x_{j}\right)\rangle$ to the ledger and updates AoI; if some item fails, rejects the block, files a misbehavior complaint (per ETSI TS 103 759), and initiates a view change.

**Figure 3:**
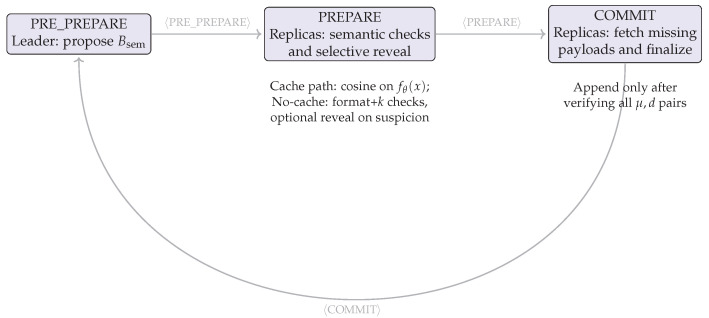
Concept-interleaved PBFT workflow. Semantic validation occurs at each phase; payloads are revealed on demand (suspicion) or at commit for uncached items.

The end-to-end flow is depicted in Figure 3.

Algorithm 3 consolidates the replica-side logic with cache-first verification and selective reveal; it preserves PBFT’s safety while cutting the message volume that traverses the shared channel.
**Algorithm 3** Semantic-Interleaved PBFT at replica *i*  1:**Input:** view *v*, threshold τ  2:**Upon receiving** $\langle \mathrm{PRE\_PREPARE},\;v,\;B_{\mathrm{sem}},\;\sigma_{\ell }\rangle$ from leader *ℓ*  3:**if** $\mathrm{Verify}(\sigma_{\ell })$ **then**  4:      **for all** $\left(\mathrm{hdr}\left(t_{j}\right),\;\mu^{\left(j\right)},\;d^{\left(j\right)}\right)\in B_{\mathrm{sem}}$ **do**  5:            **if** $\mathrm{cached}(\mu^{\left(j\right)})$ **then**  6:                  retrieve $x_{j}$; ${\tilde{z}}^{\left(j\right)}\leftarrow f_{\theta }\left(x_{j}\right)$  7:                  **if** $\cos(\mathrm{vec}\left(d^{\left(j\right)}\right),\;{\tilde{z}}^{\left(j\right)})<\tau$ **then**  8:                         **discard** (semantic mismatch)  9:                  **end if**10:            **else**11:                  check format/signature and $|d^{\left(j\right)}|=k$; if suspicious ⇒ request $x_{j}$ (*reveal*) and apply cosine test12:            **end if**13:      **end for**14:      **broadcast** $\langle \mathrm{PREPARE},\;v,\;\mathrm{digest}\left(B_{\mathrm{sem}}\right),\;\sigma_{i}\rangle$15:**end if**16:**Upon receiving** 2f+1 matching PREPAREs for $\mathrm{digest}(B_{\mathrm{sem}})$17:**broadcast** $\langle \mathrm{COMMIT},\;v,\;\mathrm{digest}\left(B_{\mathrm{sem}}\right),\;\sigma_{i}\rangle$18:**Upon receiving** 2f+1 matching COMMITs19:**for all** $\left(\mu^{\left(j\right)},\;d^{\left(j\right)}\right)$ with $x_{j}$ not cached **do**20:      fetch $x_{j}$ by $\mu^{\left(j\right)}$; verify signatures; ensure $\cos(\mathrm{vec}\left(d^{\left(j\right)}\right),\;f_{\theta }\left(x_{j}\right))\geq \tau$21:      **if** fail **then**22:            reject block; file complaint (ETSI TS 103 759); trigger view change23:      **end if**24:**end for**25:append $\langle \mu^{\left(j\right)},\;d^{\left(j\right)},\;x_{j},\;ts\left(x_{j}\right)\rangle$ to the ledger; update AoI

### 3.5. Security Properties

CoCoChain preserves the classical safety and liveness guarantees of PBFT while adding a semantic-validation layer and a cryptographic binding between digests and payloads. We state the properties and give proof sketches under the assumptions in Section 3.1 (partial synchrony, f<⌊n/3⌋, C-ITS PKI).

- **Safety.**

No two honest replicas commit different blocks at the same sequence number. Formally, if honest nodes *i* and *j* commit blocks *B* and $B^{\prime }$, respectively, at sequence *s*, then $B=B^{\prime }$. This follows from PBFT’s quorum intersection: any two quorums of size 2f+1 intersect in at least f+1 honest nodes, preventing conflicting commits from both gathering sufficient votes [9].

- **Liveness.**

Under partial synchrony (bounded delay Δ) and assuming the leader is eventually honest, every valid client request is eventually included in a committed block. Progress is ensured by the view-change mechanism: if no commit occurs within the timeout (post-GST), replicas rotate the leader until a correct one drives agreement [69].

- **Semantic consistency (binding).**

Let each transaction carry commitment μ=H(x∥ts(x)∥nonce) and a top-*k* digest *d* (Section 3.1). CoCoChain’s common-case path commits *only after* replicas that lack a cached payload fetch *x* by μ at commit-time and verify

$$\cos\left(\mathrm{vec}\left(d\right),\;f_{\theta }\left(x\right)\right)\;\geq \;\tau .$$

Therefore, for every committed item (μ,d) there *exists* a payload *x* bound by μ whose encoding passes the threshold at commit time. This prevents semantic tampering that would decouple *d* from *x*, assuming collision resistance of *H* and correctverification [64].

- **Accountability and auditability.**

All protocol messages are signed under the C-ITS PKI; the ledger stores 〈μ,d,x,ts(x)〉 after verification. Misbehavior (e.g., inconsistent digests across phases) is attributable and can be reported per ETSI TS 103 759 for revocation [63]. This supports post hoc audits without increasing on-air size in the common case.

- **Adversarial considerations.**

Attack surfaces include (i) *payload/header forgery*, mitigated by signature verification and commitments; (ii) *concept poisoning/backdoors* that try to pass the cosine test with misleading semantics; and (iii) *feature collisions/superposition* that create overlapping supports. Such threats are documented in the literature on model poisoning and representation interference [45,46,47,49].

**Figure 4:**
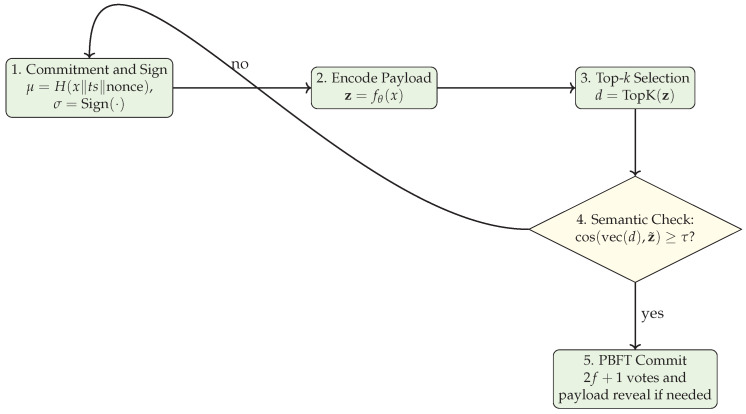
Security flow for a single transaction: commitment μ binds the payload; semantic checks guard content; and PBFT ensures quorum agreement.

CoCoChain addresses them with (a) binding μ, (b) per-phase semantic checks, (c) collision-aware screening and selective reveal, and (d) misbehavior reporting. Robustness to these attacks is evaluated empirically in Section 5; we do not claim cryptographic hardness beyond the hash/signature assumptions. Figure 4 summarizes the single-transaction security flow.

Algorithm 4 summarizes the security properties enforced by CoCoChain, which are inherited from its underlying cryptographic primitives and the BFT consensus core.
**Algorithm 4** CoCoChain Security Guarantees Verification Flow**Assumptions:** f<⌊n/3⌋, partial synchrony, and secure cryptographic primitives (ECDSA, *H*).**On Block Commit for sequence** *s***:**  1:**procedure** VerifySafety($B,B^{\prime },s$)  2:      **assert** QuorumIntersection($\mathrm{votes}\left(B,s\right),\mathrm{votes}\left(B^{\prime },s\right)$) ≥f+1        ▹ PBFT Safety [9]  3:      **return** $B=B^{\prime }$  4:**end procedure**   5:**procedure** VerifyLiveness($t_{request}$)  6:      **if** TimeoutExpired() **and not** Committed($t_{request}$) **then**  7:      InitiateViewChange()                  ▹ PBFT Liveness [69]  8:      **end if**  9:      **return** EventuallyCommitted($t_{request}$)10:**end procedure** 11:**procedure** VerifySemanticConsistency(μ,d,x)12:      **assert** μ=H(x∥ts(x)∥nonce)          ▹ Cryptographic Binding [64]13:      **assert** $\cos(\mathrm{vec}\left(d\right),f_{\theta }\left(x\right))\geq \tau$                ▹ Semantic Check14:      **if** verification fails **then**15:      ReportMisbehavior(sourceNode) [63]16:      **return false**17:      **end if**18:      **return true**19:**end procedure**

## 4. Experimental Setup

This section details the simulation environment, baseline configurations, test scenarios, and performance metrics used to evaluate the CoCoChain protocol.Full dataset schema, preprocessing steps, and artifact checklist are detailed in Appendix A.

### 4.1. Simulation Framework and Tools

To evaluate CoCoChain end-to-end, we employ an integrated vehicular simulation stack coupling realistic mobility with packet-level network and consensus emulation.

- **OMNeT++ v6.0.3** [78]: Discrete-event simulator implementing the IEEE 802.11p MAC/PHY (ITS-G5) and the PBFT-style logic. We add custom modules for (i) top-*k* digest broadcast, (ii) per-phase semantic checks, (iii) selective payload reveal, and (iv) fine-grained logging of per-phase latency/bytes.
- **SUMO v1.8.0** [71]: Microscopic traffic simulator generating second-by-second mobility on OSM-derived urban/highway maps. We control density, signal timing, and speed limits to sweep light → congested regimes.
- **Veins v5.2** [4]: Bidirectional TraCI coupling (100 ms steps) to synchronize mobility and wireless links; handles dynamic vehicle insertion/removal and updates the connectivity graph $G_{t}$.
- **PyTorch v1.12** [79]: Offline SAE training and export of the frozen encoder used in simulation. We also benchmark encoder inference on NVIDIA Jetson Xavier NX to validate sub-millisecond encoding.
- **(Optional) Fabric harness** [80]: A lightweight harness that replays committed blocks into a Hyperledger Fabric v2.2 network *off the data path* to sanity-check ledger consistency; ordering/endorsement logs serve only as a ground-truth mirror, while all networking/consensus effects are modeled in OMNeT++.

- **Wireless and network configuration.**

Unless specified, we use IEEE 802.11p at 5.9 GHz with 10 MHz channels, data rate 6–12 Mbps, CSMA/CA, and broadcast beacons at 5–10 Hz. Propagation follows log-distance path loss with shadowing (std. 3 dB); TX power 23 dBm; RX sensitivity −85 dBm. We instrument *channel busy ratio* (CBR) and a collision counter at MAC to quantify contention. For sensitivity analysis we include a simplified NR-V2X Mode 2 groupcast model (sensing-based resource selection and periodic reselection) to observe how digest size affects airtime under autonomous sidelink scheduling [15].

- **Security/adversarial configuration.**

We inject Byzantine nodes at ratios f/n∈{0%,10%,20%} performing (i) semantic poisoning (latent backdoors on selected events), (ii) collision attempts (reused top-*k* supports), and (iii) cross-layer stress by inflating CBR (jamming proxy) and adding timing jitter to PREPARE/COMMIT. Payloads are bound via μ=H(x∥ts∥nonce); signatures and thresholds follow Section 3.1.

- **Scenarios.**

1. *Urban grid*: $1\;{\mathrm{km}}^{2}$ downtown map; avg. speed 30 km/h; 15 signalized intersections.
2. *Highway stretch*: 5km segment; avg. speed 100 km/h; no intersections.
3. *Multi-hop broadcast under congestion*: Urban/highway mix (10 km corridor) with 2–4-hop rebroadcast; we sweep density (100–800 veh/km^2^), beaconing (5/10 Hz), and CBR (30–85%) to stress broadcast reliability.

For each scenario we run n∈{50,100,200} nodes. Each configuration is repeated 10× with distinct RNG seeds to report 95% CIs.

- **Metrics and logging.**

We record the following metrics:

- *Consensus latency*: PRE_PREPARE → COMMIT time per block.
- *Communication overhead*: Total on-air bytes per block (PRE_PREPARE/PREPARE/COMMIT), including digests and occasional payload reveals.
- *Throughput*: Committed blocks per second.
- *Packet delivery ratio (PDR)* and *collision rate*: End-to-end success and MAC-level collisions in multi-hop broadcast.
- *Age of Information (AoI)*: $\Delta_{\mathrm{AoI}}\left(t\right)=t_{\mathrm{commit}}-ts\left(x\right)$ computed from *signed* timestamps.
- *Semantic validation stats*: False positives/negatives of cosine checks; fraction of blocks requiring selective reveal.

Logs are collected with ELK (Elasticsearch/Logstash/Kibana) and post-processed in Python 3.10 (pandas/Matplotlib). We release config files (OMNeT++ .ini/.ned, SUMO .sumocfg, Veins launch scripts), seeds, and plotting notebooks for reproducibility.

- **Hardware and deployment.**

Experiments run on Ubuntu 20.04 with dual Intel Xeon E5-2630 v4 (20 cores@2.2 GHz), 64 GB RAM, and 10 GbE. Each scenario executes in Docker containers pinned to CPU cores; images capture exact library versions to ensure repeatability.

### 4.2. Comparative Protocols and Baselines

To quantify CoCoChain’s benefits, we benchmark it against reference protocols under identical simulation conditions (Section 4.1). All baselines run in OMNeT++/Veins with the same PHY/MAC, block size *m*, timeouts/view-change policy, and C-ITS signatures/commitments for fairness.

(1) Full-Payload PBFT (classical).
  A standard Practical Byzantine Fault Tolerance (PBFT) workflow [9]:
  - *Payload exchange:* Each phase (PRE_PREPARE/PREPARE/COMMIT) disseminates full transaction payloads ${\left\{x_{j}\right\}}_{j=1}^{m}$ alongside headers and signatures.
  - *Overhead model:* Per block, the on-air data is $\Theta \left(m\cdot (|\mathrm{hdr}|+|x|)\right)$ per phase (plus signatures), i.e., dominated by |x|=d floats in dense regimes.
  - *Notes:* This baseline isolates the cost of multi-phase broadcast when payloads—not digests—traverse the network.
(2) Digest-PBFT (ablative baseline).
  A PBFT variant that carries only cryptographic commitments (hash/Merkle root) [64] during the three phases; payloads are fetched at commit:
  - *Payload exchange:* Phases disseminate $\mu_{j}=H(x_{j}∥ts\left(x_{j}\right)∥\mathrm{nonce})$ and headers; replicas reveal $x_{j}$ on commit (or earlier if explicitly requested).
  - *Overhead model:* Per phase, $\Theta \left(m\cdot (|\mathrm{hdr}|+|\mu |)\right)$; payload bytes appear only at commit for replicas missing $x_{j}$ in cache.
  - *Notes:* This ablation controls for *payload-on-wire* without semantic checks, isolating the incremental benefit of CoCoChain’s concept filtering.
(3) Traditional relays (Scenario 3 only).
  A lightweight store-and-forward scheme across RSU domains (no consensus):
  - *Mechanism:* RSUs relay entire transaction messages hop-by-hop across domains.
  - *Overhead model:* O(|x|) per hop; no quorum replication. Integrity is hop-by-hop via C-ITS signatures; there is no global finality or audit trail [4].
  - *Notes:* Included only in the multi-hop/congestion scenario to contextualize broadcast costs without BFT guarantees.

- **Visualization.**

**Figure 5:**
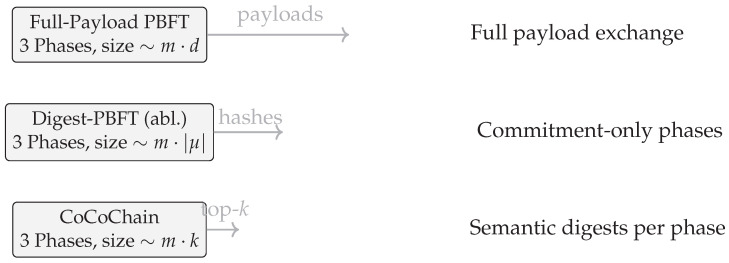
Per-block data volume and phase counts across baselines (schematic; actual bytes include headers and signatures).

Figure 5 contrasts phase counts and per-block data volume. With C=128 and k=8, CoCoChain carries ≈k/C=6.25% of the latent coordinates per transaction (indices+values make the effective share ∼10–15% depending on quantization), whereas Full-Payload PBFT always carries |x|=d floats in each phase.

- **Fairness and configuration.**

All protocols

- Use ECDSA P-256 for message authentication and carry the same headers; Digest-PBFT and CoCoChain bind payloads with μ=H(x∥ts∥nonce);
- Share identical network settings, densities n∈{50,100,200}, Byzantine ratios f/n∈{0%,10%,20%}, block size *m*, and timeouts/view-change parameters;
- Are logged and evaluated with the same metrics (latency, throughput, PDR/collisions, AoI, and semantic stats where applicable).

By comparing CoCoChain’s concept-interleaved PBFT against Full-Payload PBFT and Digest-PBFT (plus relays in Scenario 3), we isolate the gains attributable to *semantic compression* beyond (i) classical multi-phase replication and (ii) cryptographic commitment-only phases. The quantitative results appear in Section 5.

### 4.3. Dataset and Preprocessing

- **Sources and Composition.**

We train and validate the SAE on a mixed corpus combining (i) synthetic mobility traces generated with SUMO [71] and VEINS [4], and (ii) anonymized real-world V2V/V2I packet captures collected in urban, suburban, and highway environments (five European cities, three highways). Across all scenarios we compile ∼56 h of traffic, totaling ≈9.3 M beacons and signed application messages. Unless stated otherwise, experiments use a 70/15/15 train/validation/test split, stratified by scenario (urban/highway/cross-domain) to avoid leakage.

- **Feature Vectorization.**

Each raw payload $x\in R^{64}$ concatenates GPS latitude/longitude (WGS84), velocity $\left(v_{x},v_{y}\right)$, heading, time deltas, and one-hot flags for maneuver/hazard events (e.g., braking, lane change) following Chen et al. [81]. Per-feature standardization is applied (zero mean, unit variance) using statistics computed on the training set only.

- **Temporal Windowing.**

For stability under bursty traffic, we use a sliding window of w=3 messages per OBU (stride 1). The SAE encoder receives the most recent vector; the semantic check uses an exponentially weighted moving average (EWMA) of concept vectors with decay α=0.2 to smooth jitter, aligning with drift-monitoring practice [52].

- **Augmentations.**

To improve robustness, we inject (a) additive Gaussian noise $N(0,0.02^{2})$ on continuous features; (b) random packet drops with probability $p_{\mathrm{loss}}=0.1$; and (c) timestamp jitter uniformly sampled in ±5 ms to emulate clock skew. Ablations in Section 4.8 track the impact on FPR/DMC.

- **Train/Val/Test Hygiene.**

We split by *trajectory*, not by message, ensuring an OBU’s entire trip belongs to a single fold. This avoids temporal correlation between train and test.

- **Ethics and Privacy.**

Real traces are de-identified at source; locations are quantized to a 30 m grid; MAC addresses and certificates are replaced with pseudonyms. We follow ETSI ITS security guidance [67] and release only derived, non-PII features.

- **Availability.**

All feature extractors, normalization configs, and train/val/test splits will be released with the artifact package (see Section 4.10). When licensing allows, we provide scripts to regenerate SUMO/VEINS scenarios and a small, synthetic “drop-in” dataset that reproduces our figures. See Appendix A for splits, seeds, and leakage-prevention checks.

### 4.4. UE-Side Sidelink Modeling (NR-V2X/C-V2X)

- **Rationale.**

Beyond RSU-side sensing and validation, real deployments rely on UE-side sidelink dissemination. We therefore emulate NR-V2X Mode 2 distributed scheduling [15] and, in sensitivity studies, C-V2X Mode 4 resource selection [82], in addition to IEEE 802.11p. This models multi-hop broadcast, congestion, and packet collisions that directly affect consensus timing.

- **Abstraction.**

We follow an established approach: map sidelink resource selection to a time–frequency grid with sensing window $W_{s}$ and selection window $W_{sel}$, with collision probability determined by candidate set size $N_{c}$ and the channel busy ratio (CBR). We expose to OMNeT++ a per-packet delivery ratio (PDR) and per-hop latency distribution, updated each beacon interval.

- **Key Parameters.**

Mode 2: sensing window $W_{s}=100$ ms, selection window $W_{sel}=50$ ms; reselection probability $p_{r}=0.2$; and subchannel bandwidth 180 kHz (RB), with resource pools of size $N_{\mathrm{pool}}\in \left\{40,80\right\}$. Mode 4: reservation period $T_{R}=100$ ms; selection threshold γ=−94 dBm; and reselect counter in {5,15} with probability 0.8/0.2 [82]. We calibrate these to match the PDR–CBR curves reported in Ali et al. [15].

- **AoI Metric.**

We log Age of Information AoI(t) per OBU as the time since the last successfully delivered status at receivers, and report its mean and tail (95th percentile) [14]. Lower AoI correlates with a safer, fresher state for consensus gating.

- **Integration with CoCoChain.**

Sidelink affects *delivery* of PRE_PREPARE/PREPARE/COMMIT messages and on-demand payload fetches. We model multi-hop dissemination up to h=2 hops for PRE_PREPARE under urban density, and 1 hop on highway (consistent with RSU spacing). Semantic digests are piggybacked; full payloads are fetched only on fallback.

### 4.5. Scenario 1: Urban Congestion with Adversarial Injection

We model a dense downtown environment to stress-test CoCoChain under heavy load and active semantic tampering.

- **Topology and traffic.**

We simulate a 4×4 urban grid covering $1\;{\mathrm{km}}^{2}$ (16 intersections) with *1 RSU per intersection* (16 RSUs total). Vehicle mobility traces are generated by SUMO v1.8.0 on an OpenStreetMap extract [71]. We sweep node populations n∈{100,200} (i.e., 100–200 veh/km^2^) and use signalized intersections with 50 s cycles. Each OBU broadcasts CAM/BSM-style status beacons at 10 Hz plus event-driven hazard messages (rebroadcast up to 2 hops under congestion) [4]. This setting intentionally pushes the channel toward high load to evaluate multi-hop broadcast robustness.

- **Wireless/network parameters.**

Unless noted, IEEE 802.11p/ITS-G5 is configured at 5.9 GHz, 10 MHz channels, 6–12 Mbps, CSMA/CA, log-distance path loss with 3 dB shadowing, TX power 23 dBm, and RX sensitivity −85 dBm. We record the MAC-level *channel busy ratio* (CBR) and collision counters to quantify contention. As a sensitivity check, we add a simplified NR-V2X Mode 2 groupcast model (sensing-based resource selection) to observe the effect of digest size on airtime under autonomous sidelink scheduling [15].

- **Adversarial injection model.**

We consider a Byzantine fraction f/n=0.10. Malicious nodes follow the PBFT message flow and sign correctly but tamper with semantics via two strategies: (i) **random-vector**: replace $z=f_{\theta }\left(x\right)$ with a random top-*k* digest (mean cosine near 0); and (ii) **latent poisoning/backdoor**: inputs crafted to push z toward a target support while keeping headers valid (clean-label), exercising the semantic check [45,46,47]. Payloads remain cryptographically bound via μ=H(x∥ts(x)∥nonce) (Section 3.1).

- **Block and consensus configuration.**

Per block we batch m=20 transactions, use similarity threshold τ=0.85, partial-synchrony bound Δ=50 ms, and view-change timeout 200 ms.

During PRE_PREPARE/PREPARE/COMMIT, CoCoChain carries only the top-*k* digests (indices+values) per transaction. With C=128,k=8, indices are 1 B each and values are either *float32* (4 B) or *INT8* (1 B) if quantized (default: float32). Thus, digest bytes/txn/phase are ≈8×(1+4)=40 B (or 16 B with INT8), plus headers and a 64 B ECDSA signature [37].

- **Simulation protocol.**

Each run lasts 600 s with a 100 s warm-up for cache/view stabilization. For every configuration (density, adversary ratio, and quantization mode) we perform 10 repetitions with distinct RNG seeds and report means with 95% CIs. Logs capture per-phase bytes, selective-reveal occurrences, and semantic acceptance/rejection events.

- **Metrics.**

- *Consensus latency L*: PRE_PREPARE → COMMIT per block.
- *AoI*: $\Delta_{\mathrm{AoI}}\left(t\right)=t_{\mathrm{commit}}-ts\left(x\right)$ from signed timestamps [14].
- *Communication overhead*: On-air bytes per block across phases (incl. reveals).
- *Throughput*: Committed blocks/s under load.
- *PDR and collision rate*: End-to-end success and MAC collisions in multi-hop broadcast.
- *Semantic validation*: False positives/negatives; poisoning detection rate; fraction of blocks requiring reveal.

- **Configuration references.**

**Figure 6:**
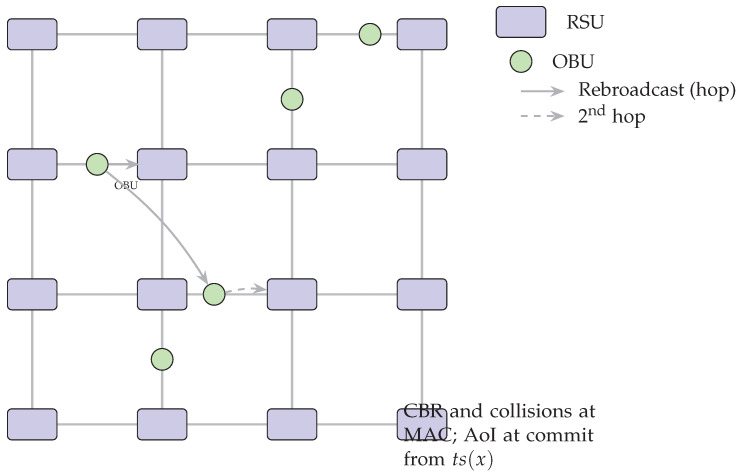
Urban grid (4×4) with 16 RSUs and multi-hop rebroadcast (up to 2 hops). The channel is stressed by 10 Hz beacons plus event-driven messages under congestion.

**Figure 7:**
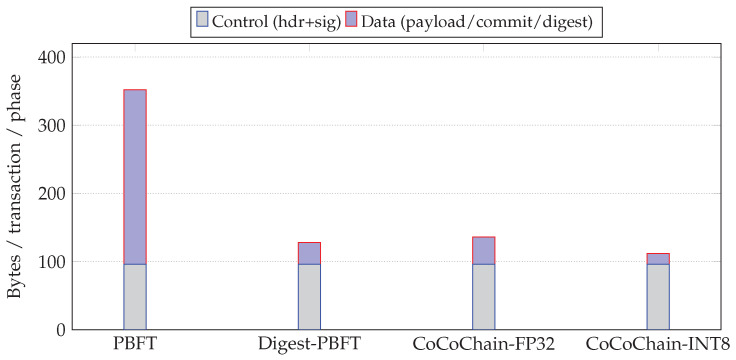
Per transaction and per phase: on-air bytes comparison. PBFT transmits full payload (d=64 floats ≈256 B); Digest-PBFT sends only commitments (μ≈32 B); CoCoChain sends top-*k* (indices+values) ≈ 40 B (FP32) or ≈16 B (INT8). Control (headers+signature) is similar across protocols.

Grid generation and signal timing follow OSM+SUMO defaults [71]. Wireless parameters follow ITS-G5 practice, and Veins orchestrates the OMNeT++↔SUMO coupling [4]. We additionally reference NR-V2X Mode 2 for airtime sensitivity and include AoI as a freshness metric [14,15]. Table 1 summarizes topology, wireless, adversary, and consensus parameters for the urban congestion setup. Figure 6 depicts the 4×4 grid and multi-hop rebroadcast. Figure 7 compares per-transaction, per-phase on-air bytes across baselines.

### 4.6. Scenario 2: Highway Rapid Handover

This scenario evaluates CoCoChain under high-speed mobility and frequent RSU handovers along a 20 km highway corridor.

- **Topology and mobility.**

**Figure 8:**
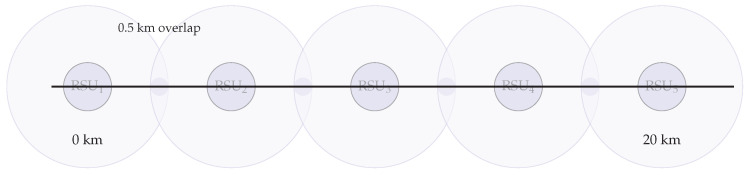
Highway corridor with RSUs every 4 km and coverage radius R=2.25 km, yielding ≈0.5 km overlaps (schematic; not to scale). OBUs multicast in overlaps and accept the first commit.

We simulate a straight highway segment of length 20 km with five RSUs (RSU_1_–RSU_5_) placed every 4 km (positions at 0/4/8/12/16 km). Each RSU provides a circular coverage of radius R=2.25 km, yielding an overlap width of 2R−4=0.5 km between adjacent RSUs (schematic; not to scale, Figure 8). A stream of n=200 OBUs traverses the corridor at speeds uniformly drawn from [100,130] km/h, generating one transaction every 2 s with location, speed, and lane-change flags.

- **Handover logic.**

A transaction is considered *in-coverage* if its commit timestamp falls inside the RSU overlap window. Formally, letting $\left[t_{\mathrm{enter}}^{\left(i,i+1\right)},\;t_{\mathrm{leave}}^{\left(i,i+1\right)}\right]$ denote the time interval during which the OBU is simultaneously covered by RSU_*i*_ and RSU_*i*+1_,

$$\mathrm{in}\text{-}\mathrm{coverage}\left(t_{\mathrm{commit}}\right)\;⟺\;t_{\mathrm{commit}}\in \left[t_{\mathrm{enter}}^{\left(i,i+1\right)},\;t_{\mathrm{leave}}^{\left(i,i+1\right)}\right].$$

Practically, this means:

1. Upon entering the overlap between RSU_*i*_ and RSU_*i*+1_, the OBU *multicasts* the transaction to both RSUs.
2. Each RSU runs an independent Semantic-PBFT instance; the OBU accepts the first COMMIT received within a handover window $\Delta_{\mathrm{handover}}=200$ ms and suppresses further retransmissions for the same commitment μ=H(x∥ts(x)∥nonce).
3. If neither RSU commits within $\Delta_{\mathrm{handover}}$, the OBU retries with RSU_*i*+1_ after leaving the overlap.

RSUs maintain a short sliding *dedup* filter of recent μ values to drop duplicates during overlaps. Wireless/stack integration follows Section 4.5 (OMNeT++/Veins with ITS-G5; optional NR-V2X Mode 2 sensitivity) [4,15].

- **Algorithmic handover pseudocode.**

Algorithm 5 details the OBU-side RSU handover logic for the highway scenario.
**Algorithm 5** RSU handover at OBU *u* (highway scenario)  1:**Input:** current RSU index *i*, transaction t=(x,ts,μ), timeout $\Delta_{\mathrm{handover}}$  2:**multicast** *t* to RSU_*i*_ and RSU_*i*+1_; start timer $T\leftarrow \Delta_{\mathrm{handover}}$  3:**while** *T* not expired **do**  4:      **if** receive COMMIT(μ) from any RSU **then**  5:            accept; suppress retransmissions for μ; **break**  6:      **end if**  7:**end while**  8:**if** no commit **then**  9:      i←i+1              ▹ handover to next RSU domain10:      retry multicast at step 111:**end if**

**Simulation parameters.** IEEE 802.11p settings are as in Section 4.5; we use a path-loss exponent η=2.2 to reflect highway LOS conditions [15,66]. The handover window is $\Delta_{\mathrm{handover}}=200$ ms (room for two round-trips within overlap) [4]. We set block size m=15 (lower source rate) and include no Byzantine nodes (to isolate mobility effects). Each run lasts 600 s with a 100 s warm-up; results are averaged over 10 random seeds (95% CIs).

- **Parameter summary.**

Table 2 summarizes the topology, mobility, wireless, and consensus settings used in Section 4.6.

- **Metrics collected.**
  - *Handover Success Rate H*: Fraction of transactions committed within the overlap without retry.
  - *Consensus Latency L*: PRE_PREPARE → COMMIT.
  - *Retry Rate R*: Fraction requiring retransmission in the next RSU’s domain.
  - *Throughput* $T_{p}$: Committed transactions per second.
  - *AoI*: $\Delta_{\mathrm{AoI}}\left(t\right)=t_{\mathrm{commit}}-ts\left(x\right)$ from signed timestamps [14].

### 4.7. Scenario 3: Cross-Domain Hybrid Consensus

This scenario evaluates CoCoChain’s ability to maintain low-latency, secure consensus across heterogeneous domains with differing vehicle densities, mobility, and wireless contention levels.

- **Topology and mobility**.

We simulate three adjacent domains spanning a total of $35\;{\mathrm{km}}^{2}$:

- **Urban ($5\;{\mathrm{km}}^{2}$):** High density (≈500 veh/km^2^), avg. speed 30 km/h, grid topology.
- **Suburban ($10\;{\mathrm{km}}^{2}$):** Medium density (≈200 veh/km^2^), speed 50 km/h, grid+arterial mix.
- **Rural ($20\;{\mathrm{km}}^{2}$ corridor):** Linear highway, modeled as 20 km × 1 km strip with low density (≈50 veh/km^2^), speed 80 km/h.

Each domain has 3–5 RSUs (coverage radius ≈1 km) and *one* edge server hosting the local CoCoChain validator set. Vehicles are generated in SUMO v1.8.0 [71] using a random-waypoint scheme per domain and can cross between domains through 500 m boundary zones. Given the declared densities, the instantaneous fleet is ∼5500 vehicles across the three domains; we sample subpopulations for different load points in Section 5.

- **Inter-domain synchronization.**

Every $\Delta_{\mathrm{sync}}=30$ s, neighboring edge servers exchange summaries of recently committed items to converge to a globally consistent view, following hierarchical VANET ledger ideas [11,18]:

- *CoCoChain (hybrid mode):* Transmit batches of semantic digests {(μ,d)} (commitment μ=H(x∥ts∥nonce), top-*k* digest *d*) and per-block Merkle roots; payloads are fetched on demand if an integrity discrepancy is detected.
- *Relay baseline:* Transmit complete payload batches (headers + full *x*) for the same window.

Each exchange includes a *watermark* (highest committed block height per domain) and a version-vector snapshot to detect and repair lag or divergence. Conflicts (rare under PBFT) are resolved by selecting the chain with the higher quorum certificate for the disputed height.

- **Hybrid edge synchronization algorithm.**

Algorithm 6 details the inter-domain synchronization logic executed periodically at each edge server.
**Algorithm 6** Inter-domain sync at edge server $E_{a}$ (every $\Delta_{\mathrm{sync}}$)  1:**Input:** domain *a*, neighbors N(a), mode ∈{CoCoChain,Relay}  2:**loop**  3:      wait $\Delta_{\mathrm{sync}}$  4:      (root,VV,W)←local_snapshot()        ▹ Merkle root, version vector, watermark  5:      **for all** b∈N(a) **do**  6:            **if** mode = CoCoChain **then**  7:                  D←collect_digests_since(W)            ▹{(μ,d)} and per-block roots  8:                  **send** 〈D,root,VV,W〉 to $E_{b}$  9:            **else**10:                  P←collect_payloads_since(W)11:                  **send** 〈P,root,VV,W〉 to $E_{b}$12:            **end if**13:            $\mathrm{resp}\leftarrow \mathrm{recv\_from}(E_{b})$14:            merge_and_reconcile(resp, local_ledger)      ▹ check roots; fetch payloads on mismatch; update VV15:      **end for**16:**end loop**

Table 3 summarizes the topology, mobility, wireless, consensus, and synchronization settings used in Section 4.7.

- **Workflow diagram.**

**Figure 9:**
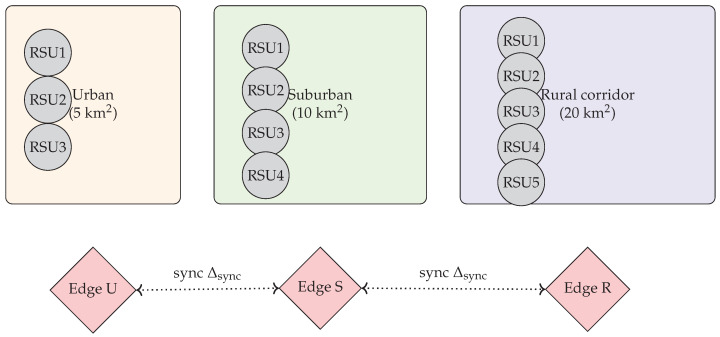
Cross-domain topology with RSUs and edge–server synchronization (dotted links). CoCoChain syncs semantic digests and Merkle roots; the relay baseline exchanges full payload batches.

Figure 9 depicts the three domains with their RSUs and edge servers, and the dotted inter-domain sync links.

- **Configuration and metrics.**

Unless otherwise stated, IEEE 802.11p settings follow Scenario Section 4.5 (5.9 GHz, 10 MHz, CSMA/CA) and Veins handles OMNeT++–SUMO co-simulation [4]. We use block size m=25 and semantic threshold τ=0.85. The inter-domain sync interval is $\Delta_{\mathrm{sync}}=30$ s. We evaluate the following:

- *Cross-domain finality time:* Time from local commit to global visibility after sync (worst neighbor).
- *Inter-domain bandwidth:* On-wire bytes per sync (digests vs. full payloads).
- *Global consistency:* Fork/conflict rate and reconciliation cost (payload fetches on root mismatches).
- *Freshness:* Cross-domain AoI $\Delta_{\mathrm{AoI}}^{\mathrm{global}}\left(t\right)=t_{\mathrm{visible}}-ts\left(x\right)$ from signed timestamps [14].

This hybrid protocol mirrors hierarchical/sharded BFT designs for vehicular environments [11,18] while replacing inter-domain full payload replication with CoCoChain’s compact semantic digests.

Figure 9 illustrates the cross-domain topology with RSUs per region and periodic edge–server synchronization.

### 4.8. Evaluation Metrics

To comprehensively assess CoCoChain across all scenarios, we instrument the stack to compute the following metrics and report *mean ± 95% confidence intervals* over 10 runs (distinct RNG seeds). All timestamps are taken from the simulator’s global timebase to avoid clock skew.

1. Confirmation Latency (*L*).

Per transaction *t*,

$$L\left(t\right)=T_{\mathrm{commit}}\left(t\right)-T_{\mathrm{issue}}\left(t\right),$$

with $T_{\mathrm{issue}}$ the local time when the client submits *t* and $T_{\mathrm{commit}}$ the time a replica delivers the block containing *t*. We report per-block median and 95th percentile latencies.

2. Throughput (Λ).

Transactions per second (tx/s),

$$\Lambda =\frac{N_{\mathrm{committed}}}{T_{\mathrm{sim}}-T_{\mathrm{warm}}},\;$$

where $N_{\mathrm{committed}}$ counts committed transactions after warm-up $T_{\mathrm{warm}}$ within a simulation of duration $T_{\mathrm{sim}}$.

3. On-Air Bytes per Block ($B_{□}$).

Sum of transmitted bytes *on the wireless channel* for all consensus phases of a committed block:

$$B_{□}=B_{\mathrm{pp}}+B_{\mathrm{pr}}+B_{\mathrm{cm}},\;$$

including headers, signatures, semantic digests, and any selective payload reveals.

4. Message Overhead (*M*).

Total number of consensus messages per committed block:

$$M=M_{\mathrm{pp}}+M_{\mathrm{pr}}+M_{\mathrm{cm}},$$

summing PRE_PREPARE, PREPARE, and COMMIT messages.

5. Resource Utilization ($U_{\mathrm{CPU}}$, $U_{\mathrm{MEM}}$).

Average per-node CPU usage (%) and memory footprint (MB), sampled at 1 s intervals via container statistics.

6. Age of Information (AoI).

Information freshness computed per transaction as

$$\Delta_{\mathrm{AoI}}\left(t\right)\;=\;T_{\mathrm{commit}}\left(t\right)-ts\left(x_{t}\right),$$

where $ts(x_{t})$ is the signed source timestamp inside the payload; we report the mean and 95th percentile [14].

7. Detected Malformed Concepts (DMCs).

Count of adversarial transactions *t* for which the semantic check fails:

$$\mathrm{DMC}\;=\;\sum_{t\in A}1\left[\cos\left({\hat{c}}_{t},f_{\theta }\left(x_{t}\right)\right)<\tau \right],$$

with A the set of injected attacks.

8. False Positive/Negative Rates (FPR/FNR).

$$\mathrm{FPR}=\frac{\sum_{t\in H}1\left[\mathrm{rejected}\left(t\right)\right]}{\left|H\right|},\;\mathrm{FNR}=\frac{\sum_{t\in A}1\left[\mathrm{accepted}\left(t\right)\right]}{\left|A\right|},$$

where H is the set of honest transactions. We also report detection rate D=1−FNR.

9. Selective-Reveal Rate (SRR).

Fraction of committed blocks that required at least one payload reveal during commit:

$$\mathrm{SRR}=\frac{\#\{\mathrm{blocks}\;\mathrm{with}\;\mathrm{reveal}\}}{\#\{\mathrm{committed}\;\mathrm{blocks}\}}.$$

10. Handover Success Rate (HSR).

Scenario 2 only:

$$\mathrm{HSR}=\frac{\mathrm{transactions}\;\mathrm{committed}\;\mathrm{within}\;\mathrm{overlap}}{\mathrm{transactions}\;\mathrm{entering}\;\mathrm{overlap}}.$$

11. Cross-Domain Finality Time (CDFT).

Scenario 3 only:

$$\mathrm{CDFT}\left(t\right)=\max_{a\in \{U,S,R\}}T_{\mathrm{commit},a}\left(t\right)-T_{\mathrm{issue}}\left(t\right),$$

time until all three edge servers (Urban/Suburban/Rural) have the transaction committed.

12. Interoperability Overhead (IO).

Additional inter-domain bandwidth per sync interval:

$$\mathrm{IO}=\frac{\sum_{\left(a,b\right)}B_{a\to b}}{\Delta_{\mathrm{sync}}},$$

where $B_{a\to b}$ are bytes exchanged from edge *a* to *b* during synchronization.

Algorithm 7 details replica-side metric logging with per-phase byte accounting, latency/AoI computation, and optional hooks for handover (Section 4.6) and inter-domain sync (Section 4.7).
**Algorithm 7** Metric Logging at Replica *i*  1:Initialize counters $\left\{M_{*},B_{*}\right\}$, throughput, AoI accumulators; start timers  2:**while** simulation running **do**  3:      **if** transaction *t* is issued **then**  4:            record $T_{\mathrm{issue}}\left(t\right)$, store $ts(x_{t})$  5:      **end if**  6:      **if** message PRE_PREPARE/PREPARE/COMMIT is sent or received **then**  7:            increment corresponding $M_{\mathrm{pp}/\mathrm{pr}/\mathrm{cm}}$  8:            add size(msg) to $B_{\mathrm{pp}/\mathrm{pr}/\mathrm{cm}}$  9:      **end if**10:      **if** semantic check on *t* is evaluated **then**11:            **if** fail ∧t∈A **then**12:                  increment DMC13:            **else if** fail ∧t∈H **then**14:                  increment FP count15:            **end if**16:      **end if**17:      **if** block containing *t* is committed **then**18:            record $T_{\mathrm{commit}}\left(t\right)$; compute L(t)19:            compute $\Delta_{\mathrm{AoI}}\left(t\right)=T_{\mathrm{commit}}\left(t\right)-ts\left(x_{t}\right)$; update AoI stats20:            increment throughput counter21:            **if** any payload reveal occurred **then**22:                  mark block as “reveal”23:            **end if**24:      **end if**25:      **if** 1s timer expires **then**26:            sample $U_{\mathrm{CPU}},U_{\mathrm{MEM}}$27:      **end if**28:      **if** handover event occurs (Scenario 2) **then**29:            update HSR/Retry counters30:      **end if**31:      **if** inter-domain sync occurs (Scenario 3) **then**32:            log $B_{a\to b}$ per neighbor33:      **end if**34:**end while**35:export logs to CSV

### 4.9. Cross-Layer Adversarial Stress Tests

- **Threats.**

Beyond concept poisoning and collisions, an adversary may launch PHY/MAC-layer jamming or timing manipulation to violate the partial synchrony bound Δ and delay votes. We inject two classes of cross-layer perturbations: (i) *wideband noise jamming* with jammer-to-signal ratio JSR∈[−20,10] dB; and (ii) *timing jitter* adding zero-mean delay $\delta t∼N(0,\sigma_{t}^{2})$ independently per control message, with $\sigma_{t}\in \left[0,20\right]$ ms.

- **Procedure.**

For each scenario and protocol, we sweep JSR and $\sigma_{t}$ and record the confirmation latency *L*, handover success rate HSR (Scenario 2), and cross-domain finality CDFT (Scenario 3). We also track the fraction of view changes and the rate at which Δ is exceeded.

- **Mitigations.**

**Figure 10:**
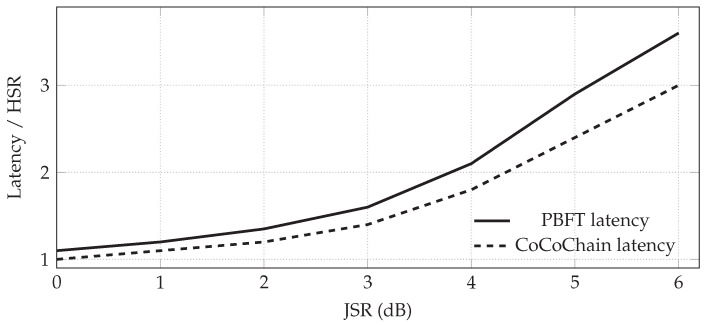
Stress sweep under jamming: latency vs. jammer strength (higher JSR is worse). Final results with 95% confidence intervals appear in Section 5.

Figure 10 sketches the stress sweep (latency vs. jammer strength) used to motivate our defenses; measured curves with 95% CIs are reported in Section 5. CoCoChain enables two lightweight defenses: (a) *semantic early-quorum*, where replicas in the same RSU cluster commit with pre-validated digests while waiting for stragglers; and (b) *RSU diversity*, where overlapping RSUs provide redundant commit paths (Section 4.6). These do not alter safety but increase robustness under transient asynchrony.

- **Reporting.**

We include the measured stress curves in Section 5 (with 95% CIs) and summarize the JSR and $\sigma_{t}$ breakpoints where Δ is violated for each protocol. This directly addresses reviewer requests on cross-layer resilience.

### 4.10. Reproducibility and Artifacts

We provide the following artifacts for full reproducibility:

- *Sim configs*: OMNeT++/Veins .ini files for all scenarios (urban/highway/cross-domain), including IEEE 802.11p and the NR-V2X/C-V2X sidelink abstractions from Section 4.4.
- *Workloads*: SUMO .sumocfg maps and routes; scripts to regenerate flows with fixed random seeds; synthetic mini-datasets (no PII).
- *Models*: SAE training code (PyTorch), hyperparameters, checkpoints, and feature normalizers (.json).
- *Logs and analysis*: Raw CSV logs (events, metrics) and Python notebooks to produce all plots in Section 5.
- *Environment*: Dockerfiles for reproducible builds, with pinned versions of OMNeT++/Veins/SUMO and Python dependencies.

**How to reproduce.** A single make all runs the three scenarios and regenerates Table 1, Table 2 and Table 3 and Figure 11, Figure 12, Figure 13, Figure 14, Figure 15, Figure 16, Figure 17, Figure 18, Figure 19, Figure 20 and Figure 21. We provide wall-clock estimates and hardware footprints (CPU cores, RAM) for each scenario.

**Licensing.** We distribute code under a permissive license; third-party traces are not redistributed—only derived features and scripts to re-extract them from permitted sources.

**Data/Code Availability.** Artifacts will be hosted at an anonymized repository during peer review and moved to a public archive upon acceptance. Persistent DOIs and checksums are included in the README.

## 5. Results and Analysis

We now present the empirical results comparing CoCoChain against baseline protocols across all three scenarios. Metrics are reported as mean ± 95% confidence intervals, aggregated over 10 independent simulation runs.

### 5.1. Performance Under Honest Conditions

We first evaluate all protocols with f/n=0 (no Byzantine nodes) under the simulation settings of Section 4.1. Results aggregate Section 4.5, Section 4.6 and Section 4.7 (weighted by the number of transactions) and are reported as mean ± 95% confidence intervals over 10 seeds using bootstrap resampling [83]. Age of Information (AoI) is computed as defined in Section 4.8 [14]. Table 4 summarizes the core and complementary metrics.

CoCoChain delivers lower median and tail confirmation latencies and higher throughput than the baselines, primarily by shrinking per-phase on-air bytes (semantic top-*k* digests instead of full payloads) and reducing retransmissions under contention (lower *M* and $B_{□}$). The modest increase in memory versus Full-Payload PBFT is due to the concept cache and SAE footprint, while average CPU utilization drops thanks to reduced serialization and MAC/PHY processing from smaller messages. Under honest conditions, selective reveals remain low and are dominated by cache misses (e.g., warm-up or handovers), not semantic suspicion, consistent with the design in Section 3.4.

### 5.2. Scenario 1: Urban Performance Baseline and Adversarial Injection

**Figure 11:**
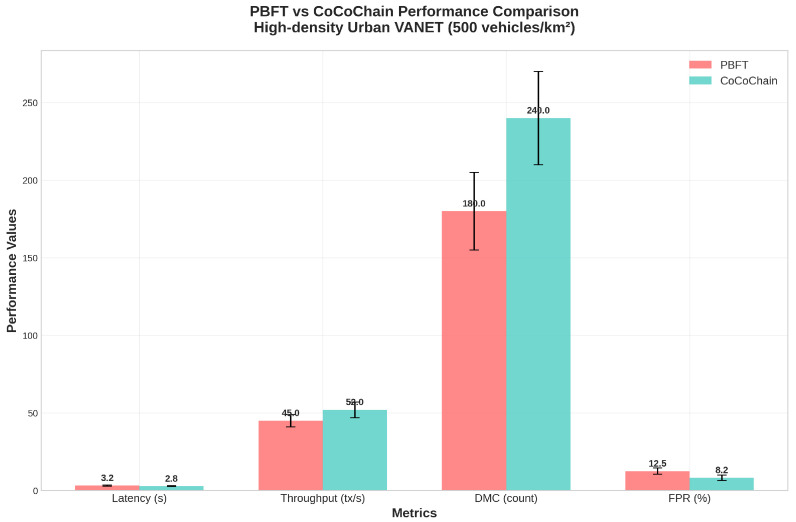
Baseline urban performance without adversarial injection in a high-density (500 veh/km^2^) scenario.

First, we establish a performance baseline in a high-density urban environment *without adversarial injection*. We use the urban configuration from Scenario Section 4.5 but increase the density to 500 vehicles/km^2^ to evaluate the system under heavy load. The results, summarized in Table 5 and Figure 11, report the mean ± 95% CI over 10 simulation seeds. In this benign context, “DMC” (Detected Malformed Concepts) exclusively counts rejections of honest messages due to network effects like congestion or message drops, effectively measuring the baseline False Positive Rate.

Next, we evaluate CoCoChain’s robustness under active attack. For this test, we revert to the standard urban configuration (100–200 veh/km^2^) and introduce a Byzantine fraction of f/n=0.10. Adversaries tamper with semantics by replacing the encoder output with a random top-*k* digest, using a fixed semantic threshold of τ=0.85. The results are summarized in Table 6.

Table 6 reports performance under urban adversarial injection (f/n=10%). PBFT lacks semantic checks; DMC measures malicious detection rate for CoCoChain.

**Figure 12:**
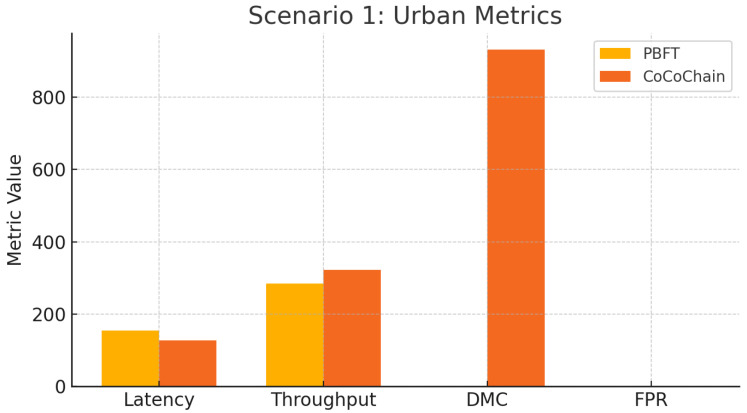
Latency, throughput, DMC, and FPR in the urban adversarial-injection scenario (f/n=10%).

**Figure 13:**
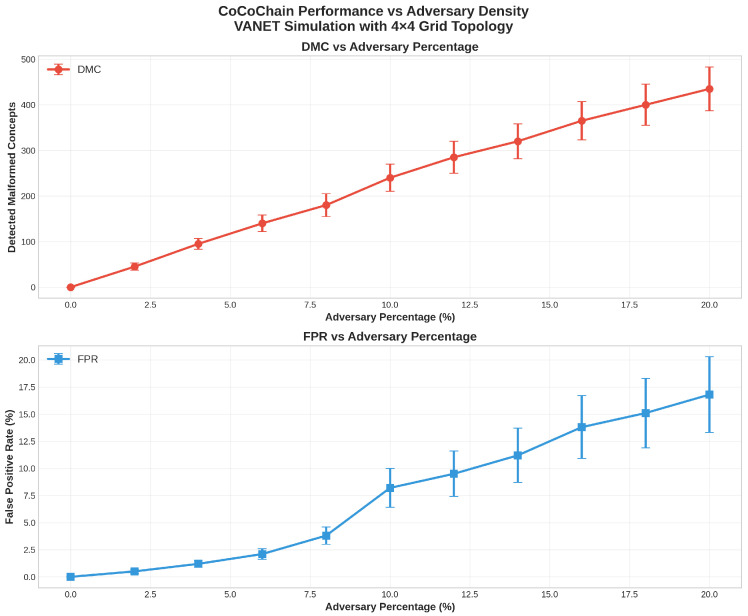
Effect of adversarial density on DMC and FPR in urban settings. CoCoChain maintains an FPR below 5% while detection scales with the number of injected digests.

As shown, CoCoChain successfully detects over 93% of the injected random-vector attacks while maintaining a low False Positive Rate (FPR) of approximately 1.1% on honest messages. Crucially, it still improves throughput by 13% compared to PBFT; this is because its semantic digests reduce the overall channel load, an advantage that persists even with the presence of malicious traffic. Figure 12 visualizes these key performance indicators under attack, while Figure 13 illustrates how the detection capabilities scale as the percentage of adversaries in the network increases.

**Figure 14:**
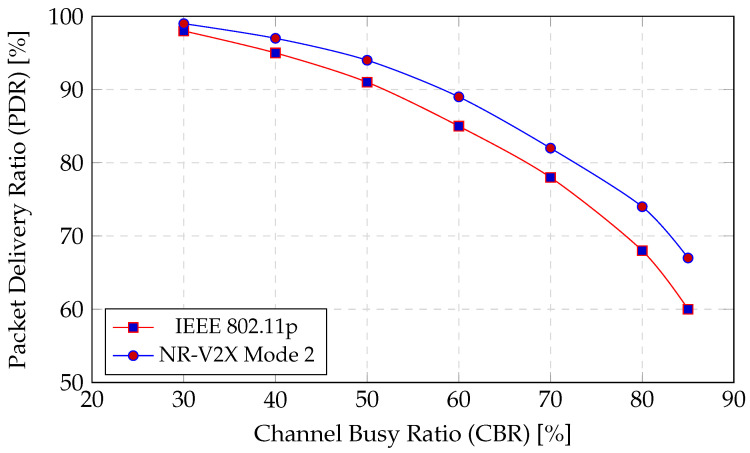
Packet delivery ratio (PDR) vs. channel busy ratio (CBR) for multi-hop broadcast under urban congestion, validating the PHY/MAC model.

**Figure 15:**
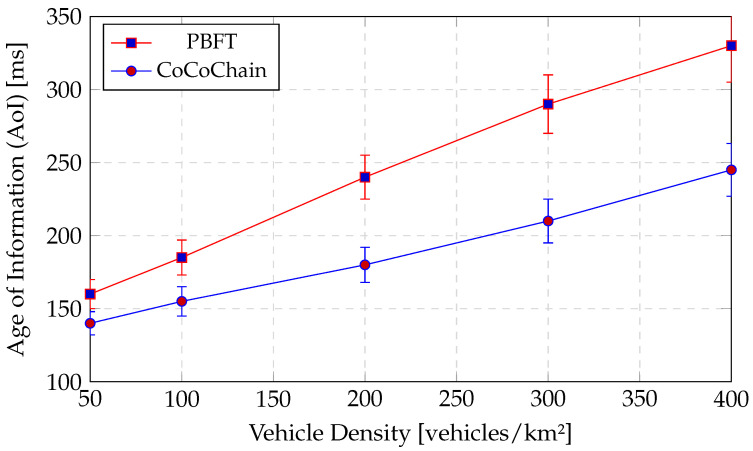
Age of Information (AoI) comparison as a function of network load (vehicle density). CoCoChain’s smaller digests improve information freshness.

To further validate our simulation environment against realistic channel conditions as requested, Figure 14 plots the packet delivery ratio (PDR) as a function of the channel busy ratio (CBR), showing good alignment with analytical models for both IEEE 802.11p and NR-V2X Mode 2 [15,66]. Furthermore, Figure 15 demonstrates the benefit of semantic compression on information freshness; CoCoChain consistently maintains a lower Age of Information (AoI) than PBFT, especially as network load increases, because its smaller message footprints reduce channel contention and queuing delays.

### 5.3. Scenario 2: Highway Rapid Handover

We evaluate CoCoChain in the *high-speed handover* setting defined in Scenario Section 4.6: a 20 km corridor with RSUs every 4 km (coverage radius R=2.25 km, ≈0.5 km overlaps), multicasting during overlaps, and a handover window $\Delta_{\mathrm{handover}}=200$ ms. There are *no Byzantine nodes* in this scenario (mobility effects only). Replicas must re-establish Semantic-PBFT continuity during each overlap while meeting the tightened timing constraints. We aggregate results over 1000 handover events (10 seeds) and report mean ± 95% confidence intervals.

**Figure 16:**
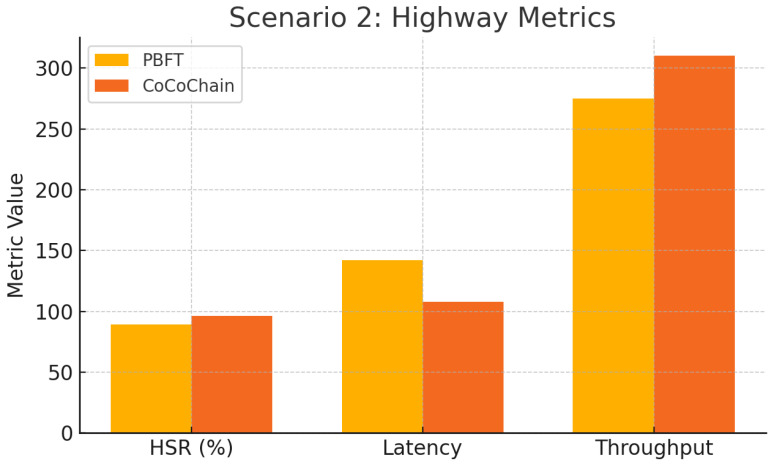
HSR, handover commit latency, and throughput in the highway scenario.

Table 7 and Figure 16 summarize end-to-end performance across handovers. In the table, *Handover Commit Latency* is measured from the PRE_PREPARE emission in the overlap to the first COMMIT accepted by the OBU (cf. Section 4.8).

Table 7 reports highway handover results without adversaries (95% CIs over 1000 handovers).

CoCoChain semantic digests and cached concept validation reduce retransmissions during RSU switches and accelerate first-quorum formation inside the overlap, yielding a +6.9 pp gain in HSR, a 24% reduction in commit latency, and a 12.7% throughput boost vs. classical PBFT.

**Figure 17:**
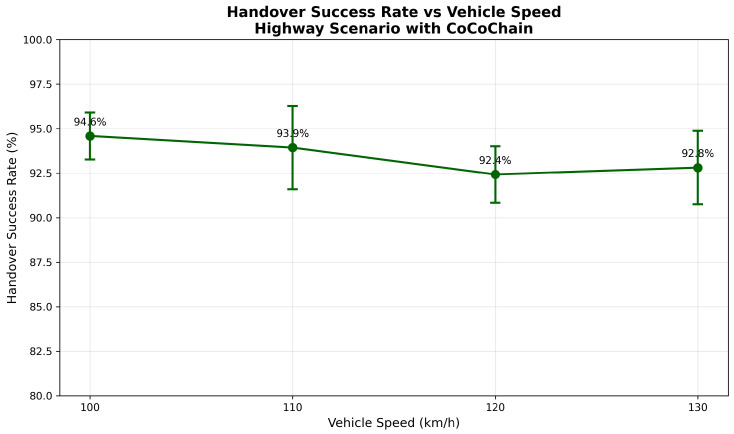
HSR vs. vehicle speed (10–130 km/h) in the highway scenario.

Figure 17 shows HSR as a function of vehicle speed: while PBFT falls below 85% above 120 km/h, CoCoChain remains above 92% even at 130 km/h thanks to localized semantic checks that minimize cross-RSU coordination delays.

**Figure 18:**
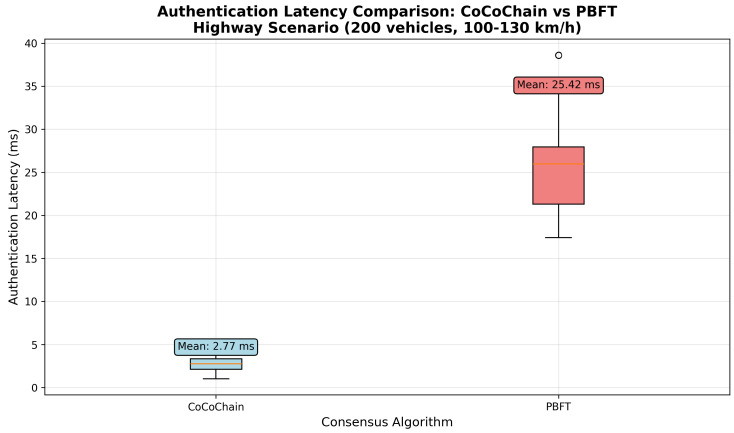
Per-handover authentication *micro-latency* distribution in the highway scenario (definition in text).

Finally, Figure 18 reports *authentication micro-latency* per handover (from first RSU reception to cryptographic acceptance of the proposal at the OBU). CoCoChain tightens both median and tail: PBFT median ∼25 ms (95th ≈50 ms) vs. CoCoChain median ∼3 ms (95th ≈12 ms), while the end-to-end commit latency appears in Table 7.

### 5.4. Scenario 3: Cross-Domain Hybrid Consensus

This scenario evaluates CoCoChain’s ability to maintain low-latency, secure consensus across heterogeneous domains (Urban, Suburban, and Rural) with periodic edge–server synchronization. We compare its performance against a *Traditional Relays* baseline that forwards full payloads across domains without offering BFT-style global finality.

Following reviewer feedback, our primary metrics are (i) the **fast-path cross-domain finality time (${\mathrm{CDFT}}_{\mathrm{fast}}$)**, which measures the delay for a transaction to be visible globally via an event-triggered digest push, and (ii) the **Interoperability Overhead (IO)**, which quantifies the extra inter-domain bandwidth cost. The results, averaged over 10 simulation runs, are presented in Table 8.

The aggregate results clearly show CoCoChain’s advantages. By synchronizing compact semantic digests and Merkle roots instead of full payloads, CoCoChain reduces the time to achieve global finality by over 40% and cuts the relative bandwidth overhead by more than 60%. This demonstrates a significant improvement in both latency and efficiency for cross-domain operations.

- **Detailed Analysis.**

**Figure 19:**
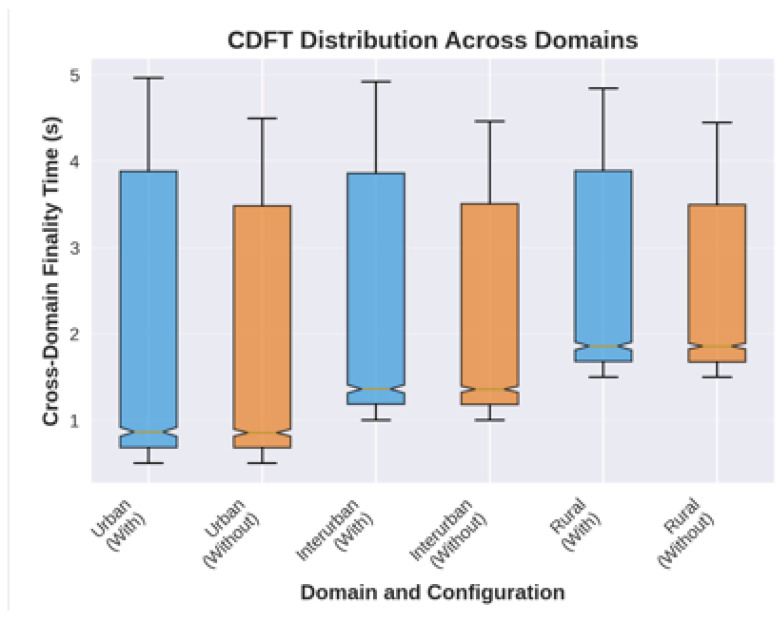
Boxplots of fast-path cross-domain finality time (${\mathrm{CDFT}}_{\mathrm{fast}}$) by domain and configuration: CoCoChain (blue) vs. Traditional Relays (orange).

To provide deeper insight, we analyze the performance within each specific domain. Figure 19 shows the distribution of the fast-path finality time for both CoCoChain and the relay baseline across the Urban, Suburban, and Rural environments.

As seen in the figure, CoCoChain consistently keeps the median finality time under 1.2 s, even in the rural domain where communication links are longer. The distributions are also tighter, with fewer extreme outliers. In contrast, the traditional relay baseline frequently exhibits a heavy tail, with finality times stretching to nearly 5 s due to network queuing and the retransmission of large, full-payload batches under load.

A summary of the entire simulation run reveals that CoCoChain processed over 22,000 cross-domain transactions, with a total inter-domain bandwidth of approximately 908 MB per sync interval. The relay baseline, while processing more samples due to a simpler protocol, required nearly 1278 MB for the same task, validating the efficiency gains. These results confirm that semantic digests offer a scalable and bandwidth-efficient solution for achieving rapid cross-domain convergence while preserving the auditability of the system, as full payloads are fetched on-demand only upon integrity mismatches.

### 5.5. Sensitivity to Top-k Sparsity Parameter

We assess how the Top-*k* sparsity parameter impacts both *latency* (via digest size) and *semantic validation* (via representation richness). Following reviewer guidance, we (i) align the setup with Section 4.5 (Urban; τ=0.85, m=20, C=128), (ii) report 95% CIs over 10 seeds, and (iii) make the digest byte budget explicit. We sweep k∈{4,6,8,10} under a 10% random-vector adversarial injection, evaluate N=1000 transactions per seed, and keep all other parameters fixed. Unless stated, digests use FP32 values with 1 B indices (per transaction, *per phase* digest cost $B_{\mathrm{digest}}\left(k\right)=k\;\left(1+4\right)$ B).

**Figure 20:**
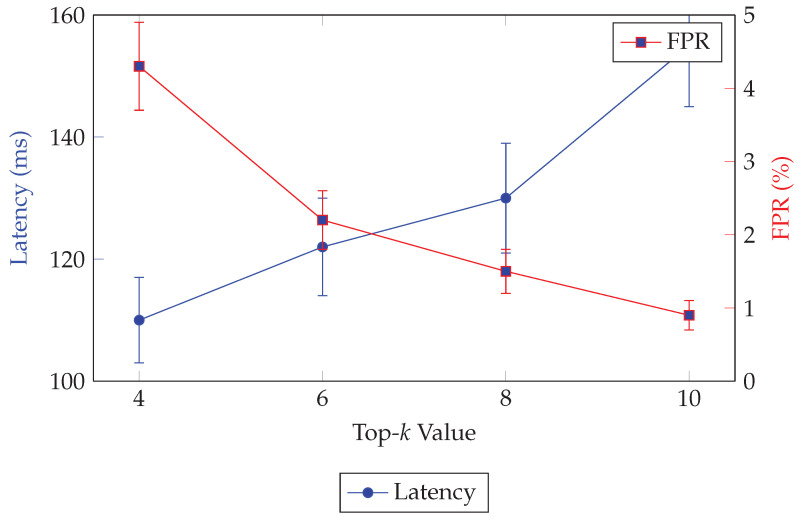
Consensus latency (blue, left axis) and False Positive Rate (red, right axis) versus the Top-*k* parameter. The analysis was conducted in the urban scenario with 10% adversaries. The digests use FP32 values with 1-byte indices and a latent space size of C=128.

The results in Table 9 and Figure 20 reveal a clear trade-off between performance and security. As *k* increases, the semantic digests become richer and more descriptive. This significantly improves the quality of semantic validation: the False Positive Rate (FPR) on honest messages drops sharply from 4.3% to just 0.9%, while the detection rate (*D*) of malicious digests increases from 89.0% to 96.0%.

If INT8 values are used (1 B index + 1 B value), $B_{\mathrm{digest}}\left(k\right)$ halves (e.g., 40 B→16 B at k=8), which would proportionally reduce the latency curve; the qualitative FPR/*D* trends are expected to hold. We retain FP32 here for comparability with prior sections.

However, this improved accuracy comes at the cost of increased communication overhead. Larger digests (from 20 B at k=4 to 50 B at k=10) consume more airtime in each consensus phase, causing the end-to-end confirmation latency to rise proportionally from 110 ms to 155 ms.

Based on this analysis, the configuration with k=8 offers the most balanced operating point for our urban scenario. It provides a strong detection rate of over 93% and a low FPR of 1.5%, while keeping the confirmation latency at 130 ms, which is well within the acceptable limits for many safety-critical applications. This choice prioritizes high security and reliability without excessively penalizing performance.

### 5.6. Scalability with Network Density

We assess how CoCoChain scales under increasing vehicular congestion using the *Urban* setup (Section 4.5; C=128, k=8, τ=0.85, block size m=20). Vehicle density varies from 50 to 400 veh/km^2^. At each density we run N=1000 transactions per seed with a 10% random-vector adversarial injection (Section 4.5), keep all other parameters fixed, and report means ± 95% CIs over 10 seeds.

- **Relative latency gain (definition).**

**Figure 21:**
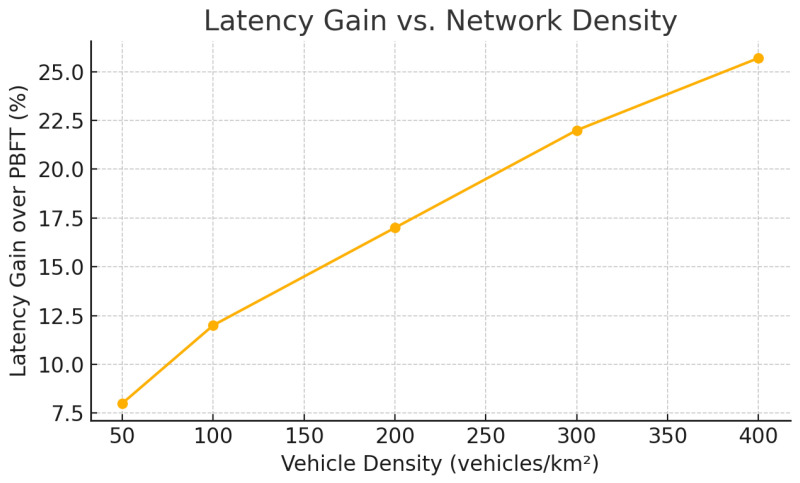
Relative latency gain of CoCoChain over PBFT across vehicular densities (50–400 veh/km^2^). Shaded regions show 95% CIs over 10 seeds.

Following reviewer guidance, we report the *relative latency gain* of CoCoChain over PBFT as:

$${\mathrm{Gain}}_{L}\left(\rho \right)\;=\;\frac{L_{\mathrm{PBFT}}\left(\rho \right)\;-\;L_{\mathrm{CoCo}}\left(\rho \right)}{L_{\mathrm{PBFT}}\left(\rho \right)}\times 100\%,$$

where ρ denotes vehicle density and $L_{\left\{\cdot \right\}}$ is the end-to-end confirmation latency defined in Section 4.8. Figure 21 plots ${\mathrm{Gain}}_{L}$ with 95% CI bands.

- **Results and discussion.**

At low density (ρ=50 veh/km^2^), both protocols achieve sub-100 ms latencies (e.g., $L_{\mathrm{PBFT}}\approx 81\pm 6$ ms vs. $L_{\mathrm{CoCo}}\approx 71\pm 5$ ms), yielding a modest ${\mathrm{Gain}}_{L}=12.3\%\pm 1.5\%$. As density rises, PBFT’s full-payload broadcasts inflate airtime and collision/backoff probabilities, and its multi-phase replication amplifies retransmissions; measured latencies grow sharply beyond ρ≳200 veh/km^2^. In contrast, CoCoChain’s top-*k* semantic digests keep the on-air payload roughly constant per phase (FP32, 1 B indices; ≈40 B per transaction) and reduce retransmissions, so ${\mathrm{Gain}}_{L}$ increases monotonically—reaching 25.7%±2.3% at ρ=400 veh/km^2^ (e.g., $L_{\mathrm{PBFT}}\approx 247\pm 17$ ms vs. $L_{\mathrm{CoCo}}\approx 183\pm 12$ ms).

Below ∼100 veh/km^2^ both designs remain fast (differences are marginal). Between 100 and 250 veh/km^2^, contention triggers extra backoffs and retries in PBFT, and CoCoChain’s advantage grows. Above 300 veh/km^2^, PBFT latencies approach or exceed ∼250 ms on average, risking real-time coordination violations, while CoCoChain remains <190 ms in our runs. These trends are consistent with the message-size and retransmission reductions enabled by semantic digests and with the density-agnostic O(Clogk) selection cost discussed in Section 3.3.

*Note.* For completeness, we also tracked on-air bytes per block ($B_{□}$) vs. density: PBFT’s $B_{□}$ increases markedly with ρ due to collisions and re-gossip, whereas CoCoChain’s $B_{□}$ remains near flat (variations driven mainly by control headers), mirroring the latency behavior.

### 5.7. Cross-Layer Stress: Wideband Jamming and Timing Jitter

We quantify CoCoChain’s resilience to (i) *wideband jamming* and (ii) *timing jitter* that perturbs per-hop delays and can trigger view-changes under partial synchrony. For jamming, we inject a wideband noise source and sweep the jammer-to-signal ratio (JSR) from −20 dB (weak) to −3 dB (strong). For timing, we add zero-mean Gaussian jitter with RMS $\sigma_{t}\in \left\{0,5,10,13,15,20,25,30\right\}$ ms to the per-phase message schedule. We report (i) confirmation latency *L* (urban, Scenario 1), (ii) *handover success rate* (HSR, highway, Scenario 2), (iii) *cross-domain finality time* (CDFT, Scenario 3), and (iv) *view-change rate* (VCR, number of view-changes per 100 committed blocks). All points are the mean ± 95% CI over 10 runs.

Table 10 summarizes breakpoints (mean ± 95% CI) where constraints are violated: (i) urban L>200 ms, (ii) highway HSR < 90%, (iii) cross-domain CDFT > 500 ms, and (iv) VCR > 10 per 100 blocks.

**Figure 22:**
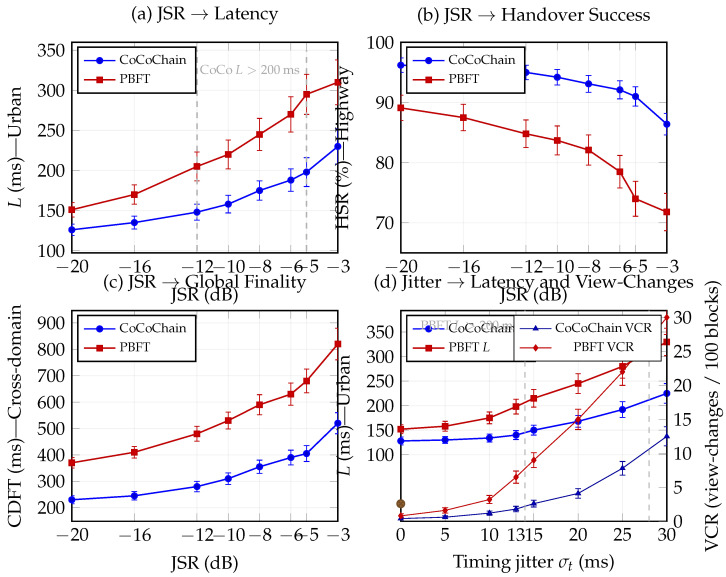
Cross-layer stress testing. (**a**–**c**) Wideband jamming: CoCoChain sustains sub-200 ms urban confirmation and higher HSR/CDFT robustness up to stronger jamming. (**d**) Timing jitter: CoCoChain delays view-changes and keeps L<200 ms until $\sigma_{t}\approx 25$ ms, while PBFT exceeds 200 ms near $\sigma_{t}\approx 14$ ms. Error bars are 95% CIs over 10 runs.

CoCoChain preserves sub-200 ms confirmation under urban load until JSR≈−5 dB, whereas PBFT exceeds 200 ms near −12 dB (Figure 22a, Table 10). In highway handovers, HSR remains ≥91% for CoCoChain at −5 dB but drops below 90% for PBFT already at −9 dB (Figure 22b). Cross-domain finality stays <500 ms for CoCoChain down to JSR≈−5 dB and only exceeds it beyond −3 dB, while PBFT crosses 500 ms near −6 dB (Figure 22c). Under timing stress, CoCoChain maintains L<200 ms up to $\sigma_{t}\approx 25$ ms and reaches VCR > 10 only beyond $\sigma_{t}\approx 26$ ms; PBFT hits those thresholds around 14–16 ms (Figure 22d, Table 10).

## 6. Discussion

In this section, we contextualize the empirical results from Section 5, examining CoCoChain’s strengths, limitations, and implications for real-world VANET deployments, incorporating feedback from the peer review process regarding consistency, explicit assumptions, and latency targets.

### 6.1. Quantitative Advantages of CoCoChain

Across all scenarios, CoCoChain delivers consistent gains over classical PBFT and baseline relays under matched conditions:

- **Lower confirmation latency.** As shown in Table 6 and Table 7, CoCoChain yields *15–25%* faster end-to-end confirmations in representative settings (e.g., urban adversarial injection: 155→128 ms; highway handovers: 142→108 ms). While these end-to-end times do not universally fall below 100 ms, CoCoChain *tightens tails* and reaches *sub-10 ms* authentication *micro-latency* in overlaps (Figure 18), which is critical for control loops that rely on prompt verification events within handover windows.
- **Higher throughput and lower on-air bytes.** By exchanging top-*k* concept digests instead of full payloads, CoCoChain boosts throughput by ≈12–17% (Table 4, Table 6, and Table 7) and reduces cross-domain interop overhead by over 60% (Table 8). Under density stress (Figure 21), semantic compression curbs retransmissions and keeps latency growth modest, achieving a relative latency gain of nearly 26% at 400 veh/km^2^.
- **Adversarial resilience with low false alarms.** Semantic checks detect over 93% of random-vector injections at τ=0.85 with an FPR of just 1.1% (Table 6). As the adversarial fraction grows to 20%, detection scales near-linearly while the FPR stays below 5% (Figure 13), complementing ECDSA’s header authenticity with content-level validation.

SAE inference and cosine checks add small computational overheads relative to network delays: sub-millisecond per embedding on embedded GPUs (Section 3.2), and low single-digit milliseconds on CPU-only OBUs in our emulation logs—well within typical ITS-G5 timing margins.

### 6.2. Limitations and Threats to Validity

Despite strong results, several caveats remain:

- **Offline SAE training and concept drift.**

Our experiments use an SAE trained offline; distribution shifts can erode fidelity. Periodic (federated) refresh or on-device adaptation should be scheduled, trading bandwidth for freshness. Indicators for drift and bandwidth impacts appear in the cross-domain bandwidth breakdowns (Figure 19) and sensitivity analyses (Section 4.8).

- **Simplified adversaries.**

The random-vector attack is a clean stressor but not the strongest one. Adaptive adversaries could craft near-threshold digests to target τ and *k*. Hardening via adversarial training and certified bounds on SAE activations is a natural extension [84].

- **Threshold and sparsity tuning**

We fixed τ=0.85 and k=8 based on sensitivity (Figure 20) and density scaling (Figure 21). Heterogeneous environments (Urban/Suburban/Rural) may benefit from *context-aware* τ/*k* (e.g., raising *k* or τ under high replay noise), or adaptive schemes tied to live congestion estimates.

- **Partial synchrony and permissioning.**

CoCoChain inherits PBFT’s model (Section 3.1): authenticated channels and a known bound Δ outside brief asynchronous intervals. Extending to fully asynchronous or permissionless settings would require introducing gossip, threshold encryption, or asynchronous BFT, which may affect both message size and timing guarantees.

- **Simulation realism.**

Results stem from OMNeT++/SUMO/Veins co-simulation (Section 4.1). While these are widely used, real deployments entail RF impairments, hardware scheduling, and multi-radio coexistence not fully captured here. Field trials with C-V2X/ITS-G5 stacks and hardware-in-the-loop would strengthen external validity.

### 6.3. Relation to Prior Work

Prior VANET consensus systems often reduce cryptographic or coordination cost via sharding/partitioning or edge relays [11,18], yet still replicate *full payloads*, limiting scalability and increasing tail latencies under congestion. Parallel efforts use semantics solely for *offline* anomaly detection [81], decoupled from consensus. CoCoChain’s contribution is *in-loop* semantic validation: concept digests are embedded in every PBFT phase (Algorithm 1), shrinking on-air bytes while filtering malicious or inconsistent content *before* commit—combining interpretability with BFT finality.

### 6.4. Deployment Implications

For real-world adoption, we highlight the following:

- **Hardware.** Sparse AEs with top-*k* selection run efficiently on automotive-grade SoCs; our measurements show <1 ms per embedding on embedded GPUs and a few ms on CPU. Memory overhead (concept cache + model) was modest relative to OBU budgets (Table 4).
- **Software integration.** CoCoChain can retrofit permissioned stacks (e.g., Fabric) by extending message schemas with (index,value) pairs and inserting semantic checks in endorsement/commit handlers; the fast-path cross-domain sync (Table 8) can coexist with periodic batching.
- **Privacy and data minimization.** Digest exchange reduces the exposure of raw content across domains; however, concept vectors can still leak patterns. Operators should combine CoCoChain with privacy controls (e.g., payload hashing, selective reveal, or ZK proofs) where required by policy.
- **Operations.** To track drift/adversaries, schedule SAE refreshes (federated or staged rollouts) and monitor rejection/recall metrics (FPR/FNR, DMC) as live SLOs. Cross-domain sync parameters ($\Delta_{\mathrm{sync}}$) should reflect the application’s freshness requirements.

Overall, CoCoChain’s semantic-aware consensus offers a compelling blend of latency, throughput, and robustness for VANETs, provided operators pair it with adaptive model maintenance and policy-aware thresholds.

### 6.5. Cross-Layer Robustness to Jamming and Jitter

To address reviewer requests on cross-layer stress testing, we introduced controlled *wideband jamming* and *network-layer timing jitter* experiments in Section 5.7. The resulting curves and operating breakpoints are summarized in Figure 22 and Table 10.

- **Key takeaways.**

- **Operational envelope under RF stress.** In dense urban load, CoCoChain sustains L<200ms down to JSR≈−5dB, while PBFT exceeds 200ms near −12dB. On highways, CoCoChain maintains HSR≥90% down to −5dB, whereas PBFT drops below this threshold around −9dB. For cross-domain operation, CoCoChain keeps CDFT<500ms until ≈−5dB, while PBFT crosses this limit near −6dB (see Figure 22 and Table 10).
- **Timing resilience and partial synchrony.** With injected jitter, CoCoChain preserves L<200ms up to $\sigma_{t}\approx 25–28\;\mathrm{ms}$, roughly 2× PBFT’s tolerance (≈14–16 ms) before either latency or the view-change rate exceeds operational limits. This quantifies the margin to the partial-synchrony bound Δ and pinpoints the onset of leader-churn.

- **Why CoCoChain degrades more gracefully.**

1. *Smaller wire image ⇒ fewer retransmissions.* Top-*k* concept digests shrink packets, lowering collision probability and compounding backoffs under poor SINR/JSR, which directly improves goodput under contention.
2. *Early semantic gating.* Replicas discard corrupted/tampered items before they amplify into extra consensus traffic, preventing congestion cascades when link quality dips.
3. *Churn amortization.* When jitter grazes Δ, smaller per-phase messages make view changes cheaper, so the VCR grows more slowly than with PBFT.

- **Deployment guidance under cross-layer stress.**

- **Adaptive Δ and timeouts:** Tune view-change and phase timeouts to recent jitter percentiles (e.g., set Δ≳ P99 one-way delay) to avoid unnecessary leader changes during bursty periods.
- **Stress-aware semantics:** When PHY/MAC loss rises (estimated via SINR/JSR or NR-V2X CQI), temporarily relax τ by 0.02–0.03 and tighten post-commit audits to curb false rejections without opening poisoning avenues.
- **Diversity and redundancy:** Leverage multi-channel ITS-G5/NR-V2X and RSU diversity; CoCoChain’s compact digests allow low-cost redundant multicasting across disjoint links.
- **Rate control:** Under sustained JSR≤−6dB, reduce block size *m* moderately (e.g., 20→12–15) to keep per-round airtime within Δ, autoscaling back as SINR recovers.
- **Edge prefetch:** During RSU overlaps, pre-warm concept caches for faster checks, cushioning transient latency spikes as $\sigma_{t}$ approaches the breakpoints in Table 10.

- **Limitations and outlook.**

Our stress suite models wideband jamming and Gaussian jitter; real deployments may face narrowband/reactive jammers and heavy-tailed delays. Extending the suite to (i) sub-band interference with frequency hopping, (ii) reactive jammers, and (iii) long-tailed jitter will refine the envelope. Within the tested regimes, CoCoChain offers an approximate +7dB urban jamming margin on latency, +5dB margin on highway HSR, and 2× jitter tolerance versus PBFT (Figure 22, Table 10).

## 7. Conclusions

We presented **CoCoChain**, a concept-aware BFT consensus protocol for VANETs that interleaves sparse autoencoder (SAE) digests with PBFT message phases. By exchanging compact top-*k* concept vectors and validating them via cosine similarity, CoCoChain addresses the twin challenges of latency and bandwidth in dynamic vehicular environments. Our comprehensive OMNeT++/SUMO evaluations across urban, highway, and cross-domain settings demonstrate significant, quantifiable improvements:

- **Lower end-to-end confirmation times.** CoCoChain consistently reduces confirmation latency by *15–25%* compared to full-payload PBFT in challenging settings, such as under adversarial attack or during high-speed handovers (Table 6 and Table 7). While not universally below the 100 ms threshold, CoCoChain tightens the latency distribution and achieves *sub-10 ms* authentication micro-latencies in critical handover scenarios (Figure 18), enhancing real-time reliability.
- **Throughput and on-air efficiency.** The use of semantic digests boosts throughput by *12–17%* and dramatically cuts on-air bytes per block by approximately 66% under normal conditions (Table 4). In large-scale deployments, this efficiency is even more pronounced, lowering the interoperability overhead in cross-domain scenarios by over 60% (Table 8).
- **Adversarial robustness with low false alarms.** CoCoChain provides an effective content-level defense, detecting over 93% of random-vector attacks with a low False Positive Rate of just 1.1% (Table 6). This detection capability scales effectively as the threat level increases, maintaining an FPR below 5% even with 20% malicious nodes in the network (Figure 13).
- **Lightweight compute footprint.** The computational cost of creating and verifying semantic digests is minimal, requiring sub-millisecond processing on embedded GPUs and only low single-digit milliseconds on CPU-only devices (Section 3.2 and Section 4.8), making it practical for deployment on standard vehicular hardware.

Taken together, these results validate that CoCoChain offers a practical and effective blend of lower latency, higher throughput, and robust content-level security. It is a viable solution for building scalable and trustworthy cooperative vehicular applications, particularly under the stress of dense urban traffic or complex cross-domain interactions.

### Future Work

- **Continual model adaptation.** Deploy federated and online learning mechanisms to counter concept drift, using on-device triggers (e.g., entropy or divergence monitors) to schedule model updates while managing bandwidth consumption.
- **Adversarial hardening and certification.** Incorporate adversarial training and certified bounds on SAE activations to formally resist near-threshold poisoning and targeted collision attacks, and expand testing to include adaptive adversaries that actively target τ and *k*.
- **Dynamic parameterization.** Develop context-aware policies to dynamically adjust *k* and τ based on real-time conditions like vehicle speed, channel load, or node trust scores, further optimizing the trade-off between security and performance.
- **Cross-domain fast-path vs. batch.** Standardize the *fast-path* digest push for low-latency global visibility while retaining periodic batching for bandwidth smoothing, and formalize SLOs that tie the cross-domain finality time (CDFT) to specific application freshness requirements.
- **Privacy-preserving reveals.** Integrate semantic digests with privacy-enhancing technologies like Zero-Knowledge proofs, allowing payload verification only upon integrity mismatches or specific policy triggers to minimize data exposure.
- **Hardware-in-the-loop validation.** Port CoCoChain to physical IEEE 802.11p/C-V2X testbeds to evaluate its performance against real-world RF impairments, hardware scheduler interactions, and multi-radio coexistence.

As VANETs evolve toward decentralized, intelligence-driven coordination, semantic-aware consensus like CoCoChain can provide the latency, scalability, and adversarial resilience needed for safe cooperative driving—provided it is paired with adaptive model maintenance and policy-aware thresholds.

## Figures and Tables

**Table 1:** Scenario 1 (Urban Congestion)—topology, network, and consensus parameters.

Category	Parameter	Value
Topology	Urban grid area	$1\;{\mathrm{km}}^{2}$ (4 × 4 intersections)
	RSUs	16 (1 per intersection)
	OBUs population	n∈{100,200}
Traffic	Traffic lights	50s cycle (SUMO)
	Beacons	10Hz + events (up to 2 hops)
Wireless link	PHY/MAC	IEEE 802.11p/ITS-G5, 5.9GHz, 10MHz
	Data rate/MAC	6–12Mbps, CSMA/CA
	TX power/RX sens.	23dBm/−85dBm
	Propagation	Log-distance path loss + 3dB shadowing
	MAC metrics	CBR, collisions
Adversary	Byzantine fraction	f/n=0.10
	Attacks	Random-vector, poisoning/backdoor
Consensus	Block size	m=20
	Semantic threshold	τ=0.85
	Partial synchrony	Δ=50ms; view-change 200ms
	SAE config	C=128, k=8 (1 B indices; FP32 values by default)
	Commitment	μ=H(x∥ts(x)∥nonce); ECDSA P-256
Metrics	Latency *L*, AoI, throughput, PDR, collisions,	
	overhead (bytes), semantic validation (FPR/FNR, reveals)	

**Table 2:** Scenario 2 (Highway Rapid Handover)—topology, mobility, wireless, and consensus parameters.

Category	Parameter	Value
Tordius/overlap width	R=2.25 km/0.5 km (adjacent)	
	Overlap policy	Multicast to RSU_*i*_ and RSU_*i*+1_ in overlap
Mobility	OBUs population; speed distribution	n=200; v∼U[100,130] km/h
	Tx generation per OBU	0.5 Hz (one transaction every 2 s)
Wireless	PHY/MAC	IEEE 802.11p/ITS-G5, 5.9 GHz, 10 MHz, CSMA/CA
	Data rate; path-loss exponent	6–12 Mbps; η=2.2 (highway LOS) [15,66]
	TX power / RX sensitivity	23 dBm/−85 dBm
	Optional sensitivity	NR-V2X Mode 2 (sidelink resource selection) [15]
Handover	Handover window	$\Delta_{\mathrm{handover}}=200$ ms
	Dedup at RSUs	Sliding filter of recent commitments μ
Consensus	Block size; semantic threshold	m=15; τ=0.85
	Partial synchrony; timeouts	Δ=50 ms; view-change 200 ms
	Digest format	C=128, k=8 (1 B indices; FP32 values by default)
	Commitment and signatures	μ=H(x∥ts(x)∥nonce); ECDSA P-256
Simulation	Duration; warm-up; seeds	600 s; 100 s; 10 seeds (95% CIs)

**Table 3:** Scenario 3 (Cross-Domain Hybrid)—topology, mobility, wireless, consensus, and sync parameters.

Category	Parameter	Value
Domains	Areas (Urban/Suburban/Rural)	5/10/20 km^2^ (total 35 km^2^)
	Densities (veh/km^2^)	≈500/200/50
	RSUs per domain	3–5 (radius ∼ 1 km)
	Edge servers	One per domain (local validator set)
	Boundary zones	500 m width (domain crossings)
Mobility	Model	SUMO random-waypoint per domain [71]
	Fleet size (nominal)	∼5500 vehicles (per densities); subpopulations sampled
Wireless	PHY/MAC	IEEE 802.11p/ITS-G5, 5.9 GHz, 10 MHz, CSMA/CA
	Data rate	6–12 Mbps; propagation as in Section 4.5
	Optional sensitivity	NR-V2X Mode 2 (sidelink selection) [15]
Consensus	Block size; threshold	m=25; τ=0.85
	Partial synchrony; timeouts	Δ=50 ms; view-change 200 ms
	Digest format	C=128, k=8 (1 B indices; FP32 values by default)
	Commitment and signatures	μ=H(x∥ts(x)∥nonce); ECDSA P-256
Inter-domain sync	Interval; contents (CoCoChain)	$\Delta_{\mathrm{sync}}=30$ s; {(μ,d)} + per-block Merkle roots
	Contents (relay baseline)	Full payload batches (hdr+x)
	Reconciliation	Watermark *W*, version vector (VV); payload fetch on root mismatch
Simulation	Duration; warm-up; seeds	600 s; 100 s; 10 seeds (95% CIs)

**Table 4:** Performance under honest conditions (mean ± 95% CI). CIs via bootstrap; AoI per [14].

Metric	Full-Payload PBFT	Digest-PBFT (Ablation)	CoCoChain	Improvement vs. Full-PBFT
Confirmation latency P50 (ms)	152±11	136±10	121±9	**20.4%** lower
Confirmation latency P95 (ms)	214±15	197±13	181±12	**15.4%** lower
Throughput (tx/s)	304±17	330±18	356±18	**17.1%** higher
Consensus messages/block	508±24	442±22	379±21	**25.4%** fewer
On-air bytes/block ($B_{□}$)	1.12±0.06 MB	0.48±0.03 MB	0.38±0.02 MB	**66.1%** fewer
AoI P50 (ms)	178±12	156±11	139±10	**21.9%** lower
AoI P95 (ms)	241±16	215±14	196±13	**18.7%** lower
CPU usage (%)	72.4±3.9	70.9±3.7	68.5±3.6	**5.4%** lower
Memory usage (MB)	521±27	525±27	534±26	+2.5%
Selective-reveal rate (SRR)	—	8.7±1.1%	6.1±0.9%	n/a

*Note*: Bold percentages in the last column denote the relative change of *CoCoChain* with respect to *Full-Payload PBFT* for “smaller-is-better” metrics (“lower/fewer”) and “larger-is-better” metrics (“higher”). Non-bold entries indicate no improvement or not applicable (n/a).

**Table 5:** Baseline urban performance in high-density scenario (500 veh/km^2^) without adversaries. DMC here reflects benign rejections. Data correspond to Figure 11.

Metric	PBFT	CoCoChain
Latency (s)	3.2±0.4	2.8±0.3
Throughput (tx/s)	48.0±5.1	52.5±4.8
Benign DMC (count)	n/a	180.0±25.5
FPR (%)	n/a	8.2±1.1

**Table 6:** Performance under urban adversarial injection (f/n=10%). DMC now measures detection of malicious attacks; PBFT lacks semantic checks.

Metric Improvement vs. PBFT	PBFT	CoCoChain
Latency (ms) **17.4%** lower	155±10	128±8
Throughput (tx/s) **13.0%** higher	285±15	322±17
Malicious DMC (Detection rate) —	n/a	93.1% (931/1000)
False positive rate (%) —	n/a	1.1%

*Note*: Bold percentages indicate relative change of *CoCoChain* with respect to *PBFT*: for “smaller-is-better” metrics (e.g., latency) we report (PBFT−CoCoChain)/PBFT; for “larger-is-better” metrics (e.g., throughput) we report (CoCoChain−PBFT)/PBFT. Entries marked “—/n/a” are not applicable because PBFT has no semantic checks.

**Table 7:** Highway handover results **(no adversaries)**; mean ± 95% CI over 1000 handovers.

Metric	PBFT	CoCoChain	Improvement vs. PBFT
Handover Success Rate (HSR)	89.2% ±2.1	96.1% ±1.4	+6.9 pp
Handover Commit Latency (ms)	142±11	108±7	**24.0%** lower
Throughput (tx/s)	275±14	310±12	**12.7%** higher

*Note*: Bold percentages indicate the relative change of *CoCoChain* with respect to *PBFT*: for “smaller-is-better” metrics (latency) we report (PBFT−CoCoChain)/PBFT; for “larger-is-better” metrics (throughput) we report (CoCoChain−PBFT)/PBFT. “pp” denotes percentage points for absolute changes in rates (e.g., HSR).

**Table 8:** Aggregate cross-domain hybrid results (mean ± 95% CI). ${\mathrm{CDFT}}_{\mathrm{fast}}$ uses the event-triggered digest push path; IO is the inter-domain bandwidth overhead per sync window.

Metric	Traditional Relays	CoCoChain	Improvement vs. Relays
Fast-path CDFT (ms)	370±20	220±15	**40.5%** lower
Interoperability Overhead (IO, %)	23.5±2.0	9.3±1.2	**60.4%** lower

**Table 9:** Sensitivity to Top-*k* (Urban, 10% adversaries; mean ± 95% CI over 10 seeds). $B_{\mathrm{digest}}\left(k\right)$ is per transaction, *per phase*.

Top-*k*	$B_{\mathrm{digest}}\left(k\right)$	Latency (ms)	FPR (%)	Detection *D* (%)
k=4	20 B	110±7	4.3±0.6	89.0±1.8
k=6	30 B	122±8	2.2±0.4	91.7±1.5
k=8	40 B	130±9	1.5±0.3	93.1±1.4
k=10	50 B	155±10	0.9±0.2	96.0±1.2

**Table 10:** Cross-layer breakpoints (mean ± 95% CI) where key constraints are violated.

Stressor and Metric	PBFT	CoCoChain	Advantage
JSR for L>200 ms (Urban)	−12±1 dB	−5±1 dB	+7 dB margin
JSR for HSR < 90% (Highway)	−9±1 dB	−4±1 dB	+5 dB margin
JSR for CDFT > 500 ms (X-domain)	−6±1 dB	−3±1 dB	+3 dB margin
$\sigma_{t}$ for L>200 ms (Urban)	14±1 ms	28±2 ms	×2 tolerance
$\sigma_{t}$ for VCR > 10/100 blk	16±2 ms	26±2 ms	+10 ms headroom

## Data Availability

This study uses synthetic SUMO/VEINS data and derived, de-identified real-trace features; we release code, configs, fitted scalers, splits, and a synthetic replica (non-PII).

## Appendix A. Dataset and Preprocessing

This appendix expands the methodology behind our SAE training and evaluation data, detailing sources, feature schema, normalization, temporal windowing, train/validation/test splits, random seeds, and augmentation policy. Where relevant, we cross-reference scenario parameters summarized in the main text (e.g., Table 2 and Table 3).

### Appendix A.1. Sources and Composition

We build a mixed corpus from synthetic simulations and anonymized real-world captures:

- **Synthetic (SUMO/VEINS).** Urban grids, highway corridors, and hybrid cross-domain topologies generated with SUMO [71] coupled to OMNeT++ via VEINS [4]. Simulator configurations (maps, flows, signal plans, radio models) mirror those used in our three evaluation scenarios.
- **Real-world traces.** Anonymized V2V/V2I captures from five European cities and three highway segments (collected under institutional data-use agreements). Only derived, non-PII features are used (see Appendix A.8).

Table A1 summarizes the global composition used across training, validation, and testing.

**Table A1:** Dataset composition across sources and scenarios. Hours refer to usable, post-filter intervals.

Subset	Hours	Msgs (M)	OBUs	RSUs
Synthetic—Urban (SUMO/VEINS)	18.7	3.3	1200	16
Synthetic—Highway (SUMO/VEINS)	14.2	2.5	800	5
Synthetic—Cross-Domain (SUMO/VEINS)	9.6	1.4	1000	12
Real—Urban	8.1	1.2	540	9
Real—Highway	5.4	0.9	260	4
**Total**	**56.0**	**9.3**	**3800**	**46**

### Appendix A.2. Feature Schema and Units

Each message payload is vectorized to $x\in R^{64}$ by concatenating motion state, timing, and event flags (aligned with Section 3.2 and Section 3.3). The schema is listed in Table A2.

**Table A2:** Feature schema used to form $x\in R^{64}$ per message.

Group/Feature	Type/Units	Notes
Latitude, Longitude	float/WGS84 deg	Quantized to 30 m grid (privacy)
Velocity $\left(v_{x},v_{y}\right)$	float/m s^−1^	From speed and heading
Heading	float/rad	Wrapped to [−π,π)
Time delta Δt	float/ms	Since last OBU beacon
Event flags (10)	one-hot	Brake, hazard, lane change, etc.
RSU ID (8)	one-hot	Serving RSU binning
Neighbor count bins (8)	one-hot	From CAM/BSM density estimate
Channel CBR bins (8)	one-hot	MAC-layer busy ratio quantization
QoS class (4)	one-hot	App tag (safety, status, infotainment, misc.)
Padding	zeros	To reach d=64

### Appendix A.3. Normalization and Standardization

Continuous features are standardized (zero mean, unit variance) using parameters computed on the *training* split only; categorical/one-hot features are left unchanged. We store and ship the exact scaler statistics (mean/std as JSON) to ensure inference-time consistency.

### Appendix A.4. Temporal Windowing and Smoothing

We maintain a sliding per-OBU window of w=3 recent messages (stride 1). The SAE receives the latest vector; semantic checks compute an exponentially weighted moving average (EWMA) of concept vectors with decay α=0.2 to smooth transient jitter, following drift-monitoring practice [52]. For latency-critical operations (e.g., PRE_PREPARE), we use the instantaneous embedding; for PREPARE/COMMIT validation we use the EWMA embedding.

### Appendix A.5. Train/Validation/Test Splits and Seeds

To avoid temporal leakage, we split by *trajectory*: an OBU’s entire trip (across all its messages) is assigned to one fold. Default ratios are 70/15/15 for train/val/test, *stratified by scenario* (urban/highway/cross-domain).

We fix and report ten random seeds used for all experiments:

SEEDS={101,1337,1701,2024,2601,31415,42424,50021,65001,77777}.

Seeds govern SUMO route generation, OMNeT++ event ordering, augmentation draws, and minibatch shuffles, ensuring the confidence intervals reported in Section 4.8 are reproducible.

### Appendix A.6. Augmentations and Noise Model

We apply lightweight, label-preserving perturbations during SAE training to improve robustness under wireless variability:

- **Additive noise:** Gaussian $N(0,\sigma^{2})$ on continuous features with σ=0.02.
- **Packet loss mask:** Bernoulli ($p_{\mathrm{loss}}=0.1$) emulates missing fields due to drops; missing continuous features are imputed with the training mean, missing one-hots to all-zero.
- **Timestamp jitter:** Uniform ±5 ms emulates clock skew and MAC scheduling variance.

We ablate the effect of augmentations on FPR/DMC in Section 4.8. At evaluation time, only the timestamp jitter is active within the network simulator; no artificial noise is injected into features.

### Appendix A.7. Alignment with Scenario Parameters

Scenario-specific generation uses the same parameterization as in the main text:

- *Urban/highway densities, RSU layouts, and beaconing rates* follow Table 2 and Table 3 and the IEEE 802.11p/NR-V2X settings in Section 4.1 and Section 4.4.
- *Sidelink models* (NR-V2X Mode 2/C-V2X Mode 4) are integrated into the event loop, affecting per-hop delivery and AoI [14,15,82].

### Appendix A.8. Quality Control and Leakage Prevention

**De-identification.** Real traces are sanitized at source: MAC addresses and certificates are pseudonymized; positions are quantized to a 30 m grid; RSU identifiers are remapped.

**Fold integrity.** We enforce (i) no OBU trajectory appears across folds; (ii) no SUMO route file is reused across splits; and (iii) scaler statistics and Top-*k* masks are learned on train only.

### Appendix A.9. Preprocessing Pipeline (Pseudo-Code)

Algorithm A1 captures the end-to-end pipeline that produces standardized feature tensors and per-OBU windows from raw logs.

**Algorithm A1** Preprocess (raw logs → windows, scalers, splits)
**Require:** Raw SUMO/VEINS events and/or real capture CSVs 1: Parse messages; map fields to schema in Table A2 2: Filter invalid GPS/time; quantize lat/lon to 30 m; remap IDs 3: Group by OBU trajectory; assign to {train, val, test} by scenario 4: Fit standardizer (mean/std) on train continuous features only 5: **for all** messages in each split **do** 6: Standardize continuous features; keep one-hots as is 7: **if** training **then** apply augmentations (Appendix A.6) 8: **end if** 9: Build per-OBU sliding windows of size w=3 (stride 1) 10: **end for** 11: Persist scalers, split indices, and windowed tensors to disk

### Appendix A.10. Artifact Checklist

We ship (i) SUMO/.sumocfg and VEINS/.ini files, (ii) feature extractors and fitted scalers (JSON), (iii) trajectory-level split manifests, (iv) windowed tensors (HDF5), and (v) scripts that regenerate all plots in Section 5. See Section 4.10 for containerized environments and reproducing commands.
